# Development of a greenhouse gas - air pollution interactions and synergies model for Korea (GAINS-Korea)

**DOI:** 10.1038/s41598-024-53632-w

**Published:** 2024-02-09

**Authors:** Jung-Hun Woo, Younha Kim, Ki-Chul Choi, Yong-Mi Lee, Youjung Jang, Jinseok Kim, Zbigniew Klimont, Dai-Gon Kim, Jae-Bum Lee, Hyungah Jin, Hyejung Hu, Young-Hwan Ahn

**Affiliations:** 1 Civil and Environmental Engineering, College of Engineering, Konkuk University, 120 Neungdong-ro, Gwangjin-gu, Seoul, 05029 Korea; 2 Department of Technology Fusion Engineering, Konkuk University, 120 Neungdong-ro, Gwangjin-gu, Seoul, 05029 Korea; 3 International Institute for Applied Systems Analysis, Schlossplatz 1, A-2361 Laxenburg, Austria; 4 Korea Environment Institute, 370 Sicheong-daero, Sejong, 30147 Korea; 5 National Institute of Environmental Research, Hwangyong-ro 42, Seogu, Incheon, 22689 Korea; 6 Department of Advanced Technology Fusion, Konkuk University, 120 Neungdong-ro, Gwangjin-gu, Seoul, 05029 Korea; 7 Department of Convergence of Climate and Environmental Studies, Sookmyung Women’s University, 100 Cheongpa-ro 47-gil, Yongsan-gu, Seoul, 04310 Korea

**Keywords:** Environmental sciences, Environmental social sciences

## Abstract

This study aimed to create Greenhouse Gas - Air Pollution Interactions and Synergies (GAINS)-Korea, an integrated model for evaluating climate and air quality policies in Korea, modeled after the international GAINS model. GAINS-Korea incorporates specific Korean data and enhances granularity for enabling local government-level analysis. The model includes source-receptor matrices used to simulate pollutant dispersion in Korea, generated through CAMx air quality modeling. GAINS-Korea's performance was evaluated by examining different scenarios for South Korea. The business as usual scenario projected emissions from 2010 to 2030, while the air quality scenario included policies to reduce air pollutants in line with air quality and greenhouse gas control plans. The maximum feasible reduction scenario incorporated more aggressive reduction technologies along with air quality measures. The developed model enabled the assessment of emission reduction effects by both greenhouse gas and air pollutant emission reduction policies across 17 local governments in Korea, including changes in PM_2.5_ (particulate matter less than 2.5 μm) concentration and associated benefits, such as reduced premature deaths. The model also provides a range of visualization tools for comparative analysis among different scenarios, making it a valuable resource for policy planning and evaluation, and supporting decision-making processes.

## Introduction

The detrimental impact of air pollutants on both human health and the environment is widely recognized. Consequently, emission standards have been established, and substantial efforts have been made to curtail emissions. Furthermore, proactive measures have been devised and implemented to mitigate the immediate effects of air pollutants on the surrounding areas. However, recent reports indicate that air pollutants exert long-term influences on the interplay between atmospheric composition and climate, thereby contributing to global climate change. Such effects encompass potential surface temperature increases and alterations in precipitation patterns^1,2^. Consequently, extensive research and international cooperation are underway to integrate climate change considerations into sustainable development and the preservation of atmospheric environments while seeking optimized solutions^3^. Moreover, reports suggest that the integration of air pollutant emission reduction policies with climate change policies targeting greenhouse gas reduction can yield considerable cost reductions and diverse additional benefits^4–7^.

However, analyzing the quantitative and qualitative effects and benefits of policies aimed at mitigating air pollution and reducing greenhouse gas emissions is a complex endeavor. It necessitates multiple procedures to scientifically examine climate change-inducing phenomena resulting from air pollution and incorporate the findings into policy establishment and evaluation. The initial step involves systematically categorizing the sources of air pollution. Subsequently, the alterations in atmospheric chemical composition caused by pollutants emitted from each source are analyzed, aiming at identifying the mechanisms and effects of long-term climate change. This process enables the establishment of effective management plans to mitigate air pollution and reduce greenhouse gas emissions originating from these sources. Furthermore, it permits the generation of vital insights for selecting efficient options by conducting cost analyses on alternatives derived from the integration of diverse air pollution and climate change policies. Given the intricate interconnections among these analytical processes, it is imperative to integrate them into a comprehensive and systematic framework.

Several studies have already developed and implemented integrated analysis models that incorporate these functionalities. A notable example is the Greenhouse Gas - Air Pollution Interactions and Synergies (GAINS) model, devised by the International Institute for Applied Systems Analysis (IIASA), which has been widely utilized as a representative tool. The GAINS model has been extensively utilized to investigate the pathways of atmospheric pollution and its environmental ramifications stemming from anthropogenic driving forces^8,9^. Numerous studies in Europe and Asia have employed the GAINS model for their research^10–16^. In China, the application of GAINS-China facilitated the evaluation of the effectiveness of industrial sector policies in the Yangtze River Delta. This study emphasized that the simultaneous implementation of air pollution control measures and greenhouse gas reduction strategies is more efficient and cost-effective, with the recognition of co-benefits being pivotal in the decision-making process for policy establishment^17^. The GAINS-JJJ model was utilized to implement and evaluate the Three-Year Action Plan for Blue Skies in the Northeast China 2 + 26 Regions, encompassing Beijing, Hebei, Henan, Shandong, Shanxi, and Tianjin^18^. The study demonstrated the synergistic effects of mitigating air pollutants and reducing greenhouse gas emissions in these regions through the policy. In South Africa, an analysis using the GAINS model was conducted to evaluate the cost–benefit implications of climate change mitigation and air pollution control policies ^19^. The study incorporated mortality and morbidity rates to calculate human health effects, particularly cardiovascular and respiratory impacts, based on air pollutant concentrations. These findings were instrumental in prioritizing policy implementation.

Korea actively pursues the establishment of robust climate change mitigation strategies and air quality management policies^20^. However, to successfully integrate and evaluate these policies, the adoption of an integrated management model, such as the GAINS model, would prove immensely advantageous. By employing such a model, it becomes feasible to analyze the synergies effects and trade-offs entailed in concomitant reductions of air pollutants and greenhouse gas emissions specific to distinct regions and emission sources. Moreover, the model facilitates the examination of technical aspects and the efficacy of pollutant mitigation measures. Through the quantitative evaluation of economic interactions, valuable analysis results can be derived, offering guidance for the development of more efficient countermeasures. Nevertheless, it is pertinent to acknowledge that applying the GAINS model directly to analyze the policies of Korea may encounter certain limitations, necessitating tailored modifications to accommodate the unique characteristics of the country.

In this study, the Greenhouse Gas - Air Pollution Interactions and Synergies Model for Korea (GAINS-Korea) was developed. The primary aim of this model is to estimate the costs of implementing emission reduction policies and assess the resulting environmental effects. This model was created based on the structure of the GAINS model, and specific features were added to suit the Korean conditions. There are three key distinctions to highlight in GAINS-Korea. First, a database was developed, encompassing activity levels, future projections, emission factors, and reduction policies specific to Korea. This comprehensive dataset serves as an input for emission forecasting. Secondly, unlike the national scope of the GAINS model, the GAINS-Korea model enables more detailed analysis at the regional level. It has been specifically designed to facilitate analysis across the 17 local governments in South Korea, providing insights into regional emission characteristics, emission reduction costs, and their effects. Thirdly, source-receptor (S-R) matrices were prepared, focusing on the Korean region, to facilitate the dispersion analysis of regional emissions. The CAMx (Comprehensive Air Quality Model with Extensions)^21^ was employed for air quality modeling (AQM), considering emissions and meteorological conditions specific to Korea. The results derived from the AQM were utilized to build the S-R matrices. Collectively, GAINS-Korea offers a comprehensive framework for evaluating climate-atmosphere policies in Korea. It leverages Korean-specific data, enables regional analysis, and incorporates an S-R matrix founded on local emissions and air quality modeling.

‘Development of GAINS-Korea’ section in this paper not only briefly outlines the fundamental structure and functionalities of GAINS-Korea but also provides an in-depth description of the research conducted to develop and customize GAINS-Korea to the specific conditions of Korea. Moving forward, “Model test scenarios” Section presents a concise summary of the analysis scenarios, and “Results and discussion” section provides their corresponding results, thereby showcasing the performance of the developed model. Finally, in ‘Conclusions’ section, the study presents the findings and conclusions derived from the research effort.

## Development of GAINS-Korea

### Structure of GAINS-Korea

**Figure 1:**
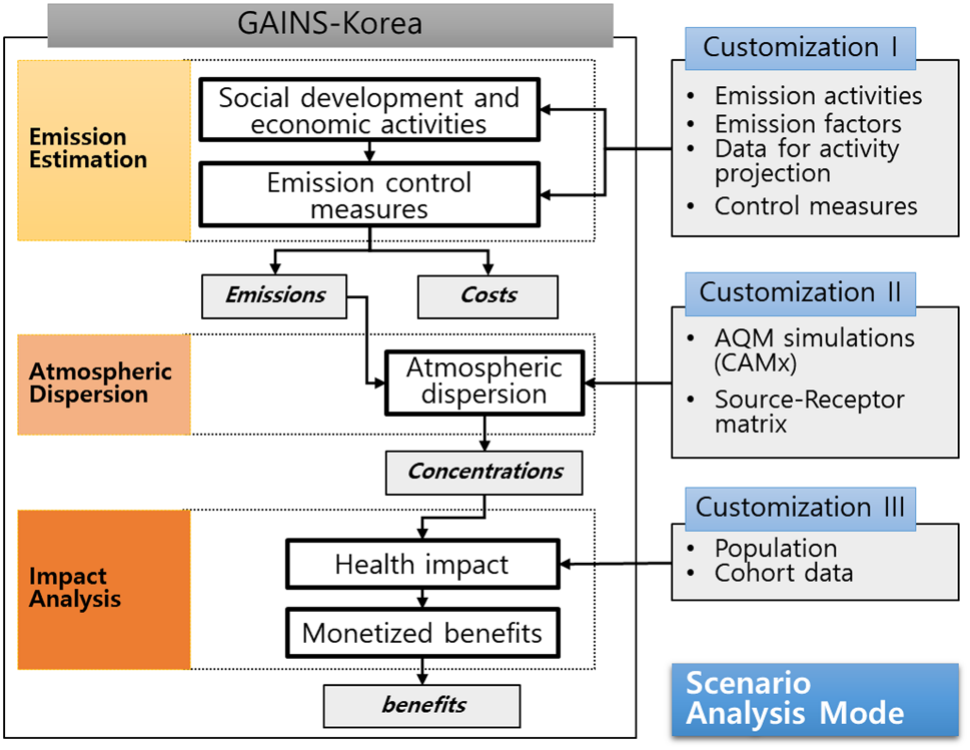
GAINS-Korea Model Structure.

The development of GAINS-Korea was based on the model structure of GAINS, with extensive efforts made to align its data structure and functions as closely as possible to facilitate future integration between the two models. Figure 1 provides a concise overview of the model structure of GAINS-Korea, where the left portion depicts the primary modules and their sequential operation, mirroring the structure of the GAINS model. A detailed description of the functions and related formulas of each module in GAINS can be found in the comprehensive work by Amann et al.^11^.

GAINS can be categorized into three main phases: emission estimation, atmospheric dispersion, and impact analysis. In the emission estimation phase, the GAINS model integrates information on future economic projections, energy and agricultural development, emission control potentials, and associated costs based on specified analysis scenarios. Using this information, future emissions and costs for reduction are estimated. Emissions of air pollutants such as sulfur dioxide (SO_2_), nitrogen oxides (NOx), ammonia (NH_3_), volatile organic compounds (VOC), and primary emissions of fine (PM_2.5_) and coarse (PM_2.5_-PM_10_) particles, along with the six greenhouse gases included in the Kyoto Protocol (carbon dioxide (CO_2_), methane (CH_4_), nitrous oxide (N_2_O), and three F-gases), are calculated using Eq. (1). The emissions estimation process considers multiple factors, namely the activity level (A_i,k_), emission factors (ef_i,k,m,p_), and the share of total activity remaining after the implementation of control measures (x_i,k,m,p_). By employing Eq. (1), emissions are systemically computed for each region, activity sector, and specific pollutant. Due to the intricate interplay between emission reduction policies and the diverse array of emission control technologies, the effectiveness of emission reduction devices exhibits variation contingent upon the specific type and scope of reduction measures implemented during the designated period. Consequently, annual emissions are computed by considering these critical factors. The quantification of annual emissions follows the formulation represented by Eq. (1):

1 $${E}_{i,p}=\sum_{k}\sum_{m}{A}_{i,k}{ef}_{i,k, m,p}{x}_{i,k, m,p}$$

where $i, k, m, and\; p$ represent the country, activity type, abatement measure, and pollutant, respectively. ${E}_{i,p}$ signifies the emissions of pollutant p (for SO_2_, NOx, VOC, NH_3_, PM_2.5_, CO_2_, CH_4_, N_2_O, among others) within country i. ${A}_{i,k}$ denotes the activity level associated with activity type k (e.g., coal consumption in power plants) within country i. ${ef}_{i,k, m,p}$ represents the emission factor pertaining to pollutant p for activity type k within country i, after the application of the specific control measure m. ${x}_{i,k, m,p}$ signifies the proportion of the total activity of activity type k within country i to which the control measure m for pollutant p is applied.

In the second part of the analysis, a dispersion analysis is conducted based on the emissions predicted in the preceding phase. To ensure effective evaluation of policy impacts, the emission source-receptor relationship for particulate matter, ozone, and their precursors is pre-calculated externally. This information is then integrated into the internal framework of the GAINS model, allowing for the prediction of concentration changes in the pollutants based on emissions. This process involves conducting multiple simulations using an Air Quality Model. To establish source-receptor (S-R) matrices, the emission source-receptor relationship is computed by considering the extent of changes in emissions and concentrations within each region.

**Figure 2:**
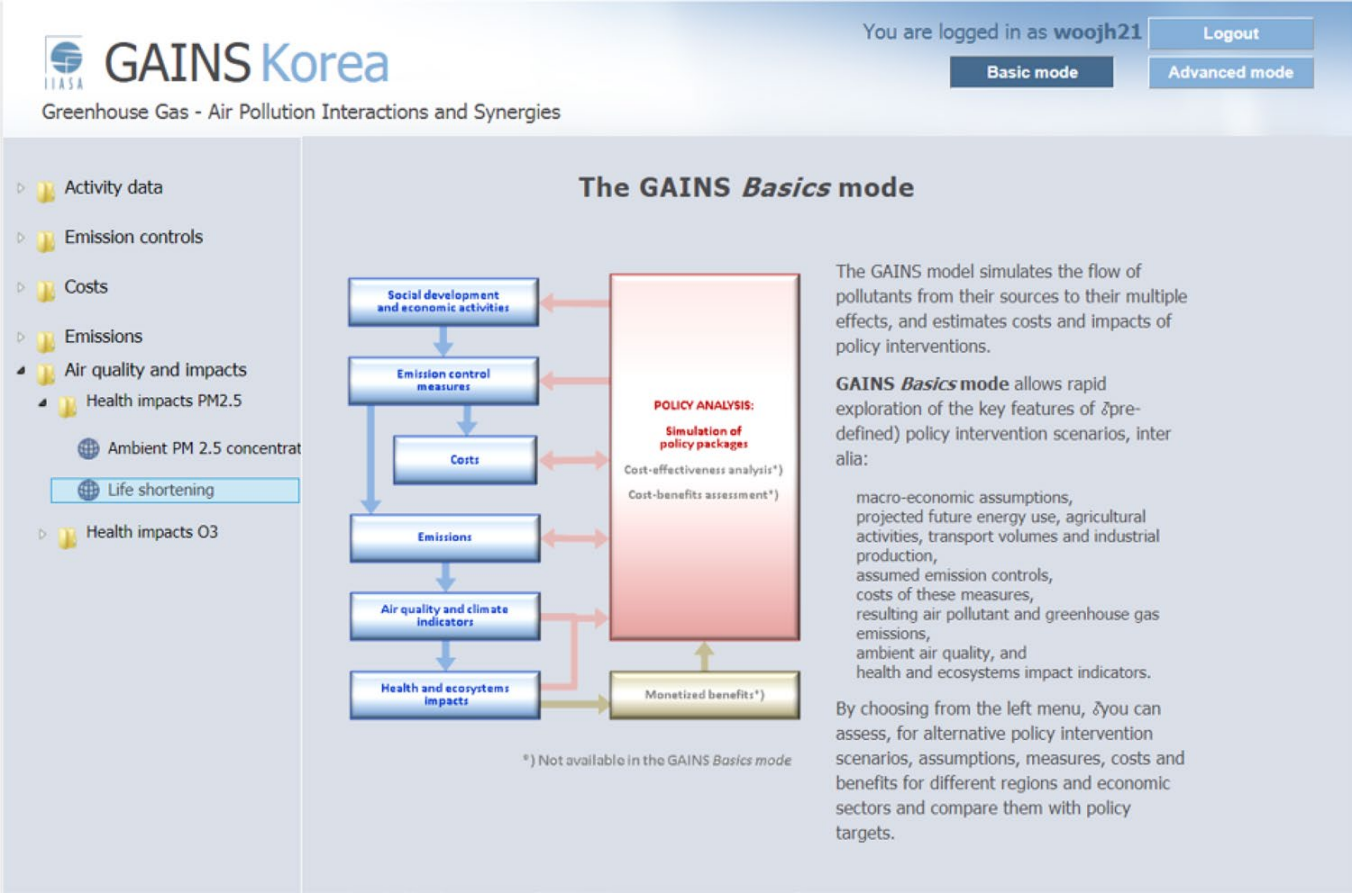
GAINS-Korea Software Website.

The third part entails the analysis of the health impacts associated with ozone and PM concentrations derived from the second phase. Subsequently, the monetary value of the reduction in human health damage is calculated. These analytical procedures are executed for each scenario, facilitating the assessment of policy effectiveness through a comparison of analysis results such as emissions, costs, and benefits. This analytical framework, known as scenario analysis mode in GAINS, is supported by a dedicated database and calculation functions that are accessible through an online-based system. Users can conveniently access this system for policy analysis and result viewing. (Fig. 2 serves as an example of the initial interface of the system).

The right side of Fig. 1 depicts externally developed components that serve as input data for the GAINS model. During the development of GAINS-Korea, specific components tailored to the Korean context are referred to as Customization I, II, and III. Customization I encompasses activity levels, emission factors, activity projections, and reduction policies, which are employed as input data for future emission estimation. Customization II involves the Source-Receptor Matrices (S-R Matrices) for atmospheric dispersion analysis. To create S-R matrices suitable for the Korean region, AQM simulations were conducted using emission and meteorological data specific to Korea. Customization III entails the integration of Korea's population and cohort data, serving as input data for health impact analysis. The subsequent section provides detailed explanations of the Koreanization process for these three components.

### Customization I: energy and emission model

GAINS-Korea employs a robust methodology to project future emissions by leveraging socio-economic forecast data and baseline year activity levels specific to the studied region. Additionally, some analysis scenarios can be established considering the reduction policies implemented in the target area, in order to calculate emission reductions and associated costs. For comprehensive analysis tailored to the Korean context, GAINS-Korea necessitates the provision of specific inputs, including activity levels, emission factors, activity projections, and reduction policies pertinent to Korea. However, due to disparities in data structures between the domestic data of Korea and the data requirements of GAINS-Korea, a meticulous mapping process was indispensable to effectively align and integrate the data.

#### Activities and emission factors mapping

In Korea, the Clean Air Policy Support System (CAPSS)^22^ provides comprehensive data on activities and emission factors for air pollutants, while the greenhouse gas inventory (GHG-CAPSS)^23^ provides information for greenhouse gases. Consequently, the activities and emission factors derived from both systems were obtained and employed to estimate air pollutant and greenhouse gas emissions for GAINS-Korea, specifically for the base year of 2010. However, due to variations in emission source and fuel classification across sectors and fuel characteristics in CAPSS, GHG-CAPSS, and GAINS, a meticulous mapping process was imperative.

Regarding the classification of emission sources, as summarized in Table 1, the activity levels and emission factor data for GAINS-Korea were generated by mapping the classification systems of CAPSS and GHG-CAPSS to align with GAINS. As for fuel mapping, bituminous coal was mapped to Brown Coal, whereas anthracite was mapped to Hard Coal. Additionally, B-A oil, B-B oil, and B-C oil were mapped to Heavy Fuel in GAINS, diesel was mapped to Medium Distillates, and fuels such as kerosene, gasoline, and similar types were mapped to Gasoline (GSL). Finally, LNG (Liquefied Natural Gas) was mapped to GAS.

**Table 1:** Mapping table for CAPSS and GHG-CAPSS to GAINS.

CAPSS	GHG-CAPSS	GAINS
Energy sector	Energy	Power plant
Domestic	Energy	Domestic
Industrial combustion	Energy	Industry
Industrial processes	Industrial processes	Industrial processes, volatile organic compounds (VOCs)
Fuel extraction	–	VOCs
Solvents	–	VOCs
Road transport	Energy	Road transport
Non_road mobile	Energy	Other transport
Waste management	Waste	Waste management
Agriculture	Agriculture	Agriculture
Other	–	Domestic
Fugitive dust	–	Domestic, industry, road transport, non-road mobile, agriculture
Biomass burning	–	Domestic, agriculture

GAINS calculates emissions from power plants, domestic sources, industry, and the transportation sector based on energy activities. Similarly, in the corresponding sectors, CAPSS and GHG-CAPSS in Korea also utilize energy activity data derived from fuel consumption, although they employ different classification systems and different units of mass or volume for calculations. Given the uncertainty associated with CAPSS activity levels and the similarity between GHG-CAPSS activity levels and the national energy statistics of Korea, GHG-CAPSS was prioritized for estimating energy activities. Conversion into calorific value was performed by the standards outlined in the energy calorie conversion standard application manual, as specified in Article 5, Paragraph 1 of the Enforcement Rule of the Framework Act on Energy.

Furthermore, CAPSS data played a crucial role in the assessment of the road transport pollutant sector, which required not only energy activity information but also the number of registered vehicles categorized by vehicle type and the total mileage covered by vehicle type (VKT). In the industrial process sector, the activity levels were determined based on product production data available in CAPSS. Moreover, for the VOC-generating industry within the industrial process sector, the production volume data from CAPSS was used to determine activity levels following the same approach employed in the industrial process sector. However, in the case of the solvents sector, where the accuracy of CAPSS may be limited, the activity levels were estimated by utilizing the emission factors from GAINS based on the emissions reported in CAPSS. In the agriculture sector, activities such as agricultural field fertilizer use, manure management, and dust scattering were derived by converting the unit of activities from CAPSS.

For the greenhouse gas emission factors, the emission factor for each major fuel category was determined by calculating a weighted average of the emission factors for the subcategorized fuel types within their respective major fuel category. Additionally, the uncontrolled emission factor was applied as the air pollutant emission factor in accordance with the Ministry of Environment's notification. However, variations in emission factors occur as CAPSS assigns multiple emission factors for each facility to a single sector in the GAINS model. To address this, representative emission factors for each sector were derived by applying the activity ratio of individual facilities as a weight to the emission factor. Nevertheless, air pollutant emissions from the energy sector in CAPSS consist of a combination of real-time emission measurements obtained from the Tele Monitoring System and activity-based emission calculations derived from national statistics. These values may differ from those generated using the GAINS methodology, which relies on activity-based calculations. For certain substances in specific sectors, adjustment factors were calculated and applied to ensure consistency between the baseline year emissions from CAPSS and GAINS-Korea. Additionally, for the road transport sector, emission factors were calculated by inversely calculating them based on the activity levels obtained from fuel consumption, since the existing emission factors in CAPSS are based on vehicle speed. In instances where emission sources were missing in the industrial process sector or the reclassified solvents sector in GAINS, emissions corresponding to these sources were included in the "OTHER_CO_2_" or "OTHER_VOC" categories, which account for other emission sources, to rectify GHG and VOC emissions.

#### Activity projection data

To estimate future activity levels, socio-economic forecast data for the region were utilized, building upon the activity data from the base year. At the national level, sector-specific and fuel-specific activity projections were then downscaled to the regional level using the approach outlined by Ahn et al.^24^. The process involved deriving growth factors for each region, sector, and fuel, which were then multiplied with the base year activity levels to estimate future activities in each region. Different approaches were employed for each sector, taking into consideration their specific characteristics. The forecasting methods for each sector are summarized in Table 2. For instance, the final energy demand forecast from the 2nd Basic Energy Plan is utilized for sectors such as industry, residential areas, commercial establishments, and transportation, while the electricity sector and energy supply sector were guided by the development plan outlined in the 6th Basic Plan for Power Supply and Demand.

**Table 2:** Source and methods for calculating growth factors.

Sector	Activity projection data	Key variables for downscaling by province	Estimation method	
Energy sector	The 6th Basic Plan for Power Supply and Demand	Power plant closure/new construction plan (Applying the location of the power plant)	Apply plan contents	
Industry	The 2nd Basic Energy Plan	Sectoral energy consumption per Gross Regional Domestic Product (GRDP)	After performing a regression analysis of GRDP per working-age population and energy consumption by sector per GRDP, the sum of local governments is proportionally adjusted to the national amount (downscaling)	
Domestic	The 2nd Basic Energy Plan	Energy consumption per capita	After performing a regression analysis on energy consumption per capita, the sum of local governments is proportionally adjusted to the national amount (downscaling)	
Transport	The 2nd Basic Energy Plan	Road (gasoline, diesel)	Energy consumption per car	Regression analysis of population per car and gasoline and diesel consumption per car then adjusting the sum of local governments in proportion to the national amount (downscaling)
		Road (other fuel)	Energy consumption per car	Use base year ratio
		Rail, airline, and maritime	Energy consumption per capita	Use base year percentage

#### Emission control measures

To assess the effects and costs of emission reduction in an analysis scenario, it is pertinent to incorporate greenhouse gas reduction policies and air pollution control policies into the GAINS-Korea system. These policies should be tailored to the scenario and cover the period from the baseline year to the target year. To operationalize these policies within GAINS-Korea, it is necessary to identify the relevant reduction technologies to each sector from the “Control strategy” dataset. Additionally, the penetration rates (Rule Penetration) for each technology should be provided in the dataset. The “Control strategy” dataset already includes a comprehensive list of reduction technologies, along with data on control efficiency and cost for each sector. Leveraging this information enables the calculation of emissions changes and associated costs resulting from the implemented policies. To incorporate the reduction technologies used in Korea into GAINS-Korea, it is imperative to input the estimated control efficiencies of these technologies in Korea into the “Control strategy” dataset. For the mobile source sector, the technologies in GAINS-Korea align with the EURO vehicle standards. Therefore, the vehicle emission standards in Korea for each model year were compared to the EURO standards, and the registration ratios of vehicles based on EURO emission standards in the baseline year were used to calculate the policy penetration rates (Rule Penetration). In the non-road mobile source sector, where information regarding the application of reduction technologies is limited, the policy penetration rates (Rule Penetration) were generated based on the “No Control” and “Not suitable for control” categories.

The GAINS model includes data for cost calculation, encompassing common scenarios across all countries. This entails considering various costs associated not only with reduction technologies, including abatement techniques, unit investment costs, and fixed and variable costs, but also additional costs related to labor, energy demand, resource requirements, and waste management. The GAINS model enables the calculation of mitigation costs by considering country-specific circumstances and technological and national environmental factors. The model also quantifies the value of societal resources allocated to emission reduction, typically assessed based on production costs rather than consumer prices. Producer or intermediary interventions that may result in price increases are excluded, and taxes included in production costs are likewise disregarded. A fundamental assumption in the GAINS cost calculation is the existence of a freely accessible market for mitigation equipment on equal terms for all countries. Consequently, capital investments for specific technologies are regarded as independent actions undertaken by implementing countries. Furthermore, the cost calculation procedure accommodates the specific conditions of particular countries, such as regional characteristics, wage levels, and emission factors. To adapt the cost calculation logic of GAINS to the domestic context, it was necessary to determine the price level of the baseline year to be used as input data. Accordingly, in order to select the appropriate baseline year price, data from 2010 to the present, with a specific focus on key factors crucial for cost estimation, such as the annual operational rates of power plants, labor costs, and investment costs associated with environmental pollution prevention facilities, were collated for Korea.

### Customization II: air quality and source apportionment model

#### AQM simulation

**Figure 3:**
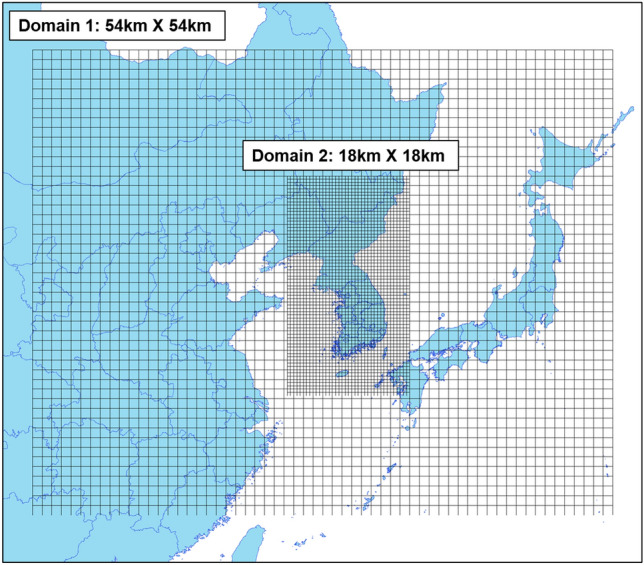
Spatial Distribution of Modeling Domains in This Study. This map was created using GIS software (ArcMap; ArcGIS Desktop; https://www.esri.com/en-us/arcgis/products/arcgis-desktop/resources). The map sources for China, Japan, and North Korea were acquired from the HDX (The Humanitarian Data Exchange) websites: China (https://data.humdata.org/dataset/cod-ab-chn), Japan (https://data.humdata.org/dataset/cod-ab-jpn), and North Korea (https://data.humdata.org/dataset/cod-ab-prk). The map source for South Korea is from the iGISMAP website (https://www.igismap.com/download-south-korea-shapefile/).

To develop source-receptor matrices for Korea, regional-scale air quality modeling was conducted. The modeling domains used in this study are depicted in Fig. 3. Domain 1, with a grid resolution of 54 × 54 km^2^, encompasses not only Korea but also key regions of China, Japan, and North Korea, thus enabling the modeling of the influence of neighboring countries. Domain 2, with a grid resolution of 18 × 18 km^2^, represents the entire Korean Peninsula and provides the appropriate resolution for generating source-receptor matrices, particularly for Korea.

To simulate atmospheric dispersion, the regional-scale meteorological model MM5 (Fifth-Generation Penn State/NCAR Mesoscale Model) was employed to replicate atmospheric flows. The emission inventory for the year 2010 used the official inventory of Korea, CAPSS. For countries in Northeast Asia, excluding Korea, the 2010 emission inventory from CREATEv1^25^ was utilized. The SMOKE-Asia (Sparse Matrix Operator Kernel Emissions-Asia)^26^ was used to incorporate these emission inventories into the input data for atmospheric modeling. The AQM system used in this study is CAMx (Comprehensive Air Quality Model with Extensions) v6.0, which enables modeling at desired spatial and temporal resolutions and incorporates the Source Apportionment technique for creating source-receptor matrices. A summary of the AQM modeling framework is provided in Table 3.

**Table 3:** AQM modeling framework.

Chemical transport model	CAMx version 6.0 with PSAT/OSAT
Chemical mechanism	CB05
Emissions	
Anthropogenic emission model	SMOKE-Asia
Emissions inventory	CAPSS 2010 (South Korea)
	CREATEv1 2010 (Other)
Meteorological model	MM5
Period	2005
Domain	Extent
	- Domain 1 (East Asia)
	- Domain 2 (South Korea)
	Grid resolution (domain)
	- 54 × 54 km^2^
	- 18 × 18 km^2^

In the AQM modeling option settings for model execution, the photodissociation rate was derived from observations obtained by the OMI (Ozone Monitoring Instrument) satellite and used as input data for the model. The initial conditions and boundary conditions concentrations for the model domain were derived from the results of a global model (GEOS-Chem) conducted by the National Institute of Environmental Research and applied as references^27^. The main settings required to run the CAMx model are summarized in Table 4.

**Table 4:** Model options applied in CAMx.

Model option	Set up	Model option	Set up
Chemical mechanism	CB05	PiG submodel	None
Probing tool	OSAT	Drydep_module	WESELY89
Advection solver	PPM	Wet deposition	True
Chemistry solver	EBI	Diffusion	ACM2

#### Source-Receptor matrix development

**Figure 4:**
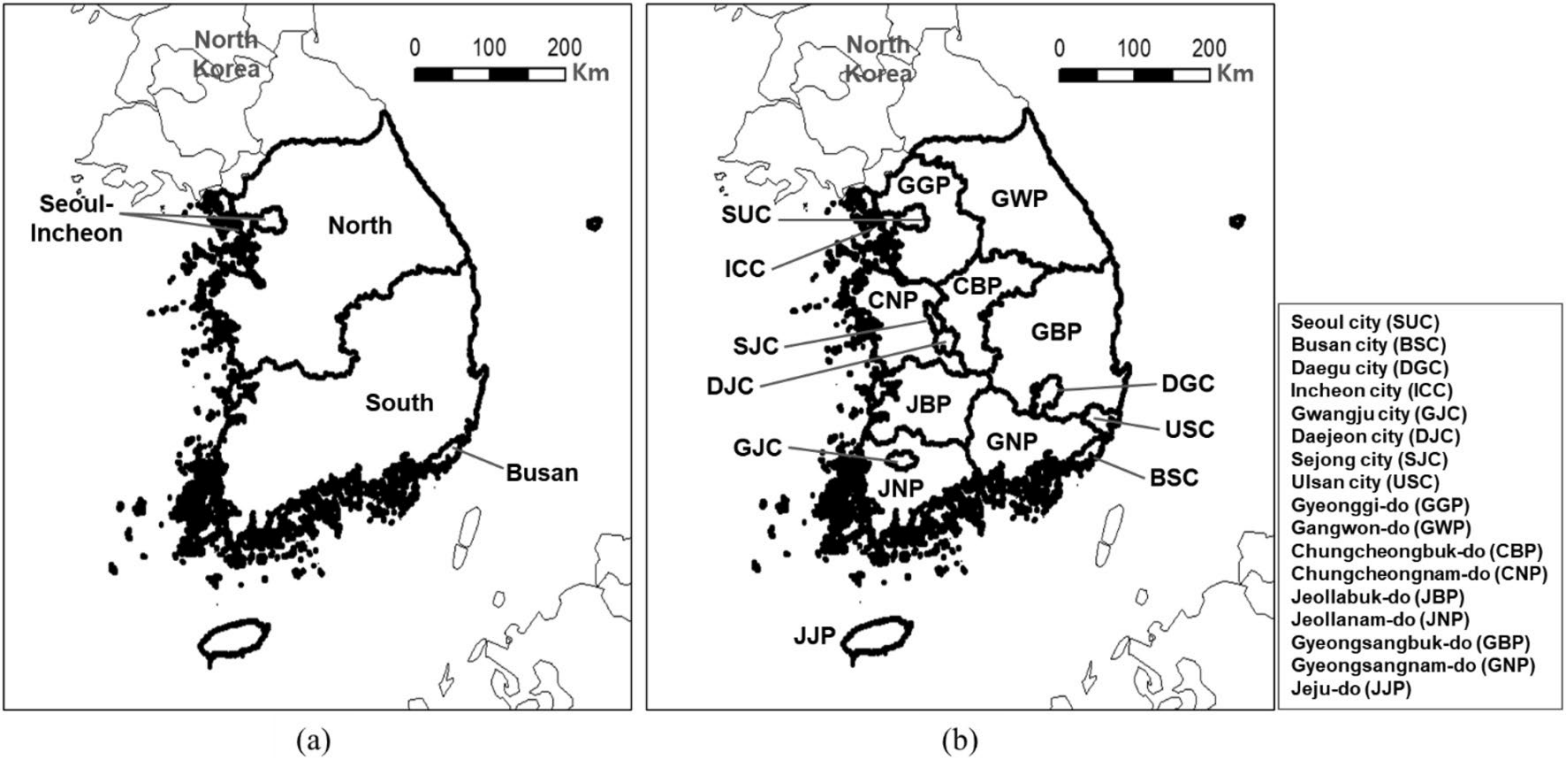
Definition of Administrative Boundaries in South Korea for GAINS (**a**) and GAINS-Korea (**b**). These maps were created using GIS software (ArcMap; ArcGIS Desktop; https://www.esri.com/en-us/arcgis/products/arcgis-desktop/resources). A portion of the map created for Fig. 3 was utilized to indicate the division of Korean regions.

As shown in Fig. 4a, the GAINS model designates only Seoul-Incheon and Busan as separate administrative regions in Korea, while the remaining areas are classified as the northern and southern regions of Korea. However, this representation inadequately captures the actual situation in Korea. Consequently, in the GAINS-Korea framework depicted in Fig. 4b, the administrative regions of Korea were divided into 17 local governments to construct source-receptor (S-R) matrices.

The source-receptor (S-R) matrices were constructed not only for particulate matter but also for ozone. For particulate matter, the developed S-R matrices included primary PM_2.5_ as well as secondary PM_2.5_. Table 5 summarizes the list of substances considered in the development of the S-R matrices, including six types of primary particulate matter and five types of secondary particulate matter.

**Table 5:** List of PM_2.5_ species in the CAMx model.

Primary PM_2.5_	Secondary PM_2.5_
Elemental Carbon (EC)	Sulfate (SO_4_)
Primary Organic Aerosol (POA)	Particulate Nitrate (NO_3_)
Crustal Fine (FCRS)	Ammonium (NH_4_)
Other Fine (FPRM)	Particulate Mercury (Hg(p))
Crustal Coarse (CCRS)	Secondary Organic Aerosol (SOA)
Other Coarse (CPRM)	–

Considering the geographical location of Korea, the impact of air pollutants emitted by neighboring countries assumes paramount significance. Therefore, the major countries within the East Asia domain were also considered source regions in assessing their contributions to receptor locations. While the contributions from external countries are not directly reflected in the source-receptor matrices of GAINS-Korea, they are accounted for as background concentrations in the model. This allows for the evaluation of contributions from China, Japan, and North Korea, alongside those from the administrative regions of Korea. A total of 20 emission regions within the East Asia domain were analyzed to assess contributions. As described above, modeling simulations were performed for the entire period of 2005 using meteorological data.

**Figure 5:**
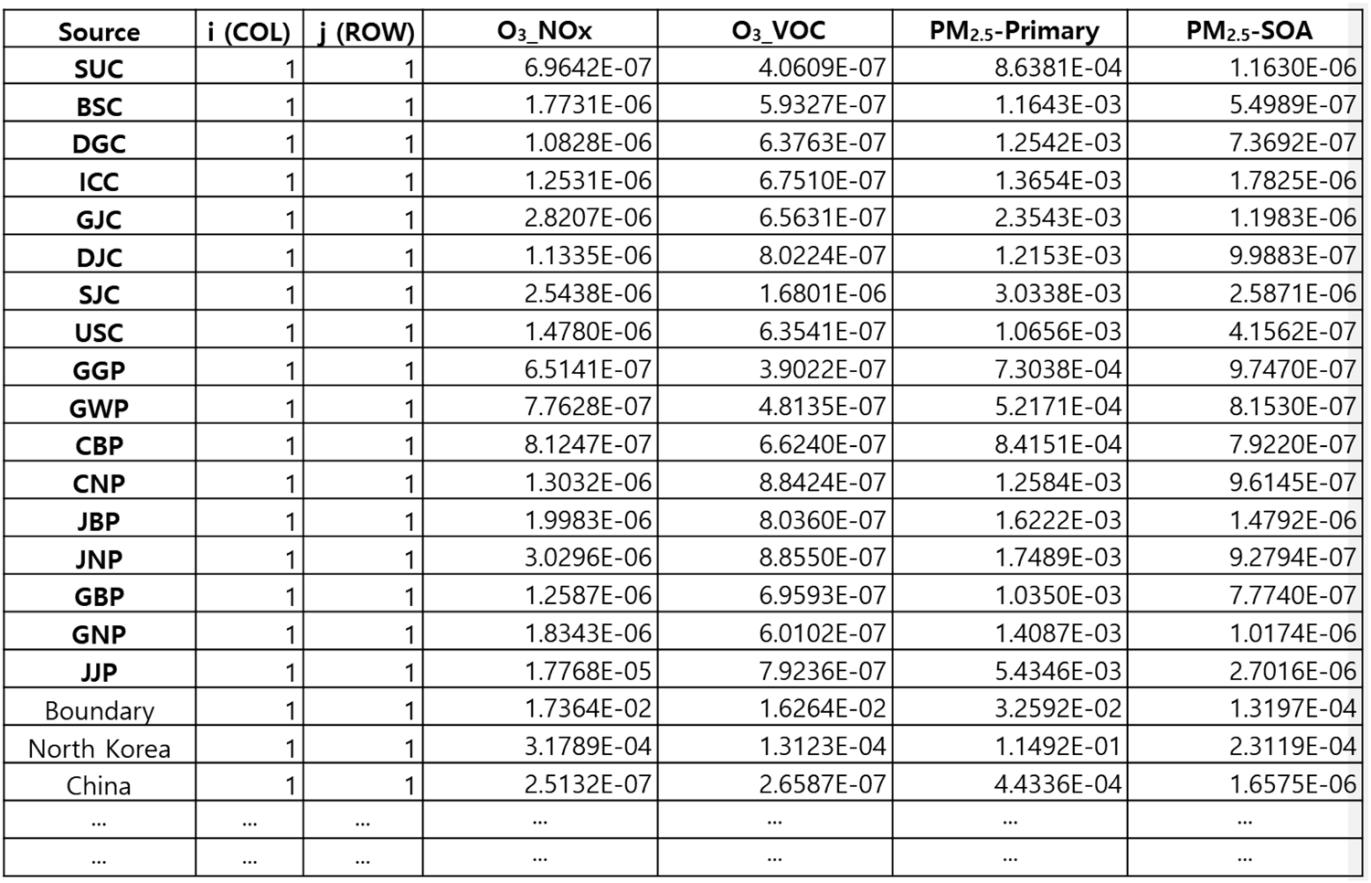
Source-Receptor Matrix for GAINS-Korea.

To develop source-receptor (S-R) matrices for Korea, a detailed grid domain centered around the Korean Peninsula was established, and region-to-grid matrices were created. Figure 5 represents parts of the results of the source-receptor matrices developed in this study. For ozone and PM_2.5_ linear transfer matrices were constructed to illustrate the contribution concentrations to each grid cell based on the unit emissions from the source regions. The zone matrices were created for each major precursor, namely NOx and VOC. The PM_2.5_ matrices were developed for primary PM_2.5_ as well as secondary organic aerosol (SOA).

The developed matrices were transformed to enable the calculation of contribution concentrations per unit emission within the GAINS-Korea model. These matrices were incorporated into the GAINS-Korea model as a fundamental module, enabling the estimation of concentration changes for each grid cell based on emission variations under different scenarios, thereby assessing the impact of dispersion and atmospheric influence. To implement this, actual data must be prepared for each grid cell in the target domain. In this study, a total of 35 × 65 grid cells were considered, with 21 regions assigned to each grid cell, resulting in a total of 45,500 data points to be calculated and incorporated into the model.

### Customization III: health impact model

In the impact analysis module of GAINS-Korea, health impacts resulting from air pollution are quantified. It allows for the quantification of health effects caused by human exposure to PM_2.5_, which is formed as secondary pollutants from the emissions of primary particulate matter, as well as SOx, NOx, and NH_3_.

Equation (2) represents the calculation for the annual mean concentration of PM_2.5_ at receptor point j. In the given equation, the emission quantities from pollution sources, ${p}_{i},{s}_{i}, {n}_{i}, {and a}_{i}$ are estimated annual emissions for each pollutant, and the ${\alpha }_{ij}, {v}_{ij}, {\sigma }_{ij},and {\pi }_{ij}$ matrices utilize the S-R matrices developed for Korea as described in ‘Customization II’ Section.

2 $${PM2.5}_{j}= \sum_{i}{\pi }_{ij}*{p}_{i}+\sum_{i}{\sigma }_{ij}*{s}_{i} +\sum_{i}{\alpha }_{ij}*{a}_{i} +\sum_{i}{v}_{ij}*{n}_{i} +{k}_{j}$$

where $$PM2.5_{j} \;{\text{represents}}\;{\text{the}}\;{\text{annual}}\;{\text{mean}}\;{\text{concentration}}\;{\text{of}}\;PM_{2.5} \;{\text{at}}\;{\text{receptor}}\;{\text{point}}\;j.$$ $$p_{i} ,s_{i} , n_{i} , a_{i} \;{\text{denote}}\;{\text{the}}\;{\text{emissions}}\;{\text{of}}\;{\text{PM}}_{2.5} , {\text{SO}}_{2} , {\text{NO}}_{{\text{x}}} , {\text{and}} {\text{NH}}_{3} ,\;{\text{resepectively}},\;{\text{at}}\;{\text{the}}\;{\text{source}}\;{\text{location}}\;i.$$ $$\alpha_{ij} , v_{ij} , \sigma_{ij} ,\pi_{ij} \;{\text{refer}}\;{\text{to}}\;{\text{the}}\;{\text{matrices}}\;{\text{containing}}\;{\text{coeffcients}}\;{\text{for}}\;{\text{reduced}}\;\left( {\upalpha } \right)\;{\text{and}}\;{\text{oxidized}}\;\left( {\text{v}} \right)\;{\text{nitrogen}},\;{\text{sulfur }}\left( {\upsigma } \right)$$ $$\mathrm{and\;primary\;PM}2.5\;\left(\uppi \right).$$ $${k}_{j}\;\mathrm{represents\;the\;backgroud\;concentration\;at\;receptor\;point}\;j.$$

The GAINS model^11^ estimates the increase in premature deaths associated with long-term exposure to PM_2.5_, focusing on the population aged 30 and above, based on findings from the American Cancer Society cohort study^28^. Similarly, in GAINS-Korea, Eq. (3) is employed to calculate changes in mortality rates linked to PM_2.5_ concentrations at receptor point j for the exposed group compared to the unexposed group among individuals aged 30 and above. To customize the model for the Korean context, GAINS-Korea incorporates regional population data, including projected population estimates and age distribution spanning from 2000 to 2030, as well as life tables specific to the Korean population.

The change in life years lived ($\Delta {L}_{l}$) for a given country (*l*) is computed using Eq. (3), which integrates various factors such as population, pollutant concentrations, and cohort-specific information. The equation captures the complex relationship between these variables and provides insights into their impact on life expectancy.

3 $$\Delta {L}_{l}= \sum_{c={w}_{0}}^{{w}_{1}}\Delta {L}_{c,j}= \beta \sum_{j\in l}{PM}_{j}\frac{{Pop}_{j}}{{Pop}_{l}}\sum_{c={w}_{0}}^{{w}_{1}}{Pop}_{c,l}{\int }_{c}^{{w}_{1}}{l}_{c}(t){{\text{log}}l}_{c}(t)dt$$

In Eq. (3), the symbols represent the following: $\Delta {L}_{l}\;\mathrm{represents\;the\;change\;in\;life\;years\;lived\;for\;country}\;l.$ ${w}_{0}: \mathrm{denotes\;the\;starting\;analysis\;time}.$ ${w}_{1}: \mathrm{represents\;the\;maximum\;age}.$ $\Delta {L}_{c,j}\;\mathrm{signifies\; the\; change\; in\; life\; years\; lived\; for\; cohort\; c\; in\; receptor\; point }j.$ $\beta$ is defined as 0.006, as given in Pope et al.^28^. ${PM}_{j}$ refers to the $\mathrm{annual\;mean\;concentration\;of}\;{PM}_{2.5}\;\mathrm{at\;receptor\;point }\;j.$ ${Pop}_{j}$ represents the total population in the $\mathrm{receptor\;point}\;j$*,* considering individuals at least of age ${w}_{0}$ = 30. ${Pop}_{l}$ signifies the total population in ${\text{country}}\;l$*,* considering individuals at least of age ${{\text{w}}}_{0}$ = 30. ${Pop}_{c,l} \mathrm{is\;population\;in\;cohort\;c\;in\;country}\;l.$ ${l}_{c}\left(t\right) \mathrm{represents\;the\;survival\;function},\;\mathrm{ indicating}$ the percentage of cohort c members alive after time* t* has elapsed since the starting time ${w}_{0}.$

## Model test scenarios

To validate the functionality of the developed model and evaluate its potential applications, a comprehensive case study was conducted. The base year selected was 2010, and the modeling period ranged from 2015 to 2030 in 5-year increments for analysis. The primary focus of this study encompassed atmospheric pollutants such as CO, NOx, SO_2_, PM, VOC, and NH_3_, while greenhouse gases consisted of CO_2_, N_2_O, and CH_4_.

Three distinct alternative scenarios were devised for the analysis, namely Business as Usual (BAU), Air Quality (AQ), and Maximum Feasible Reduction (MFR). Each scenario incorporated changes in activity levels resulting from greenhouse gas reduction policies. Moreover, the developed model incorporated emission reduction technologies that reflected variations in air pollution management policies, and control strategies for each scenario were inputted into the developed model. The analysis was conducted to compare the emissions, PM concentrations, and human health impacts among the scenarios for each pollutant.

### Scenario A: BAU (business as usual)

In the BAU scenario, emission projections were formulated for each region, fuel type, and sector from 2010 to 2030, based on energy and socio-economic outlooks, with 2010 as the reference year. This scenario assumes that the emission reduction policies implemented in 2010 will remain unchanged until 2030. The CAPSS inventory reflects the efforts of the government and local authorities in emission reduction. Therefore, the 2010 CAPSS emission data were used to evaluate the efficiency of the emission reduction policies implemented in 2010. For point sources, it is assumed that the emission reduction technologies installed in major point sources already reflect the current emission reduction policies. As for other emission sources, where specific emission reduction technologies cannot be identified, it is assumed that the reported emissions in the current CAPSS data already account for the efforts made to reduce emissions in 2010. Regarding mobile sources, it is assumed that the technology utilized in 2010 will persist until 2030, disregarding any planned emission standards for future vehicle production.

### Scenario B: AQ (air quality-air pollutant reduction)

The AQ scenario aims to assess the emission reduction effects of newly planned air quality policies that were not included in the BAU scenario. This scenario provides an evaluation of the feasibility and effectiveness of the planned policies. The AQ scenario integrates additional policies scheduled for implementation until 2030 into the BAU scenario. Policies that are challenging to quantify, such as campaigns or research support, were excluded, and only policies that can be quantified were considered. To achieve this, regional air quality policies were thoroughly examined, and a summary of the implemented policies in the model is provided in Table 6. In the case of the Seoul Metropolitan Area, particular emphasis was placed on the implementation of the "Second Improvement Plan for Air Quality Management in the Seoul Metropolitan Area" scheduled from 2015 to 2024. For other regulated areas, such as the Busan metropolitan area, Daegu metropolitan area, and Gwangyang Bay area, control measures implemented by local governments were applied as appropriate.

**Table 6:** Policies applied for the air pollutant reduction (AQ) scenario.

Policy	Pollutant	Sector
Strengthen air pollutant cap-and-trade	NOx, SOx	Power plant, industrial boilers, and furnaces
Support low-NOx burner substitution and Denitrification facility (SCR) installation	NOx	Power plant, industrial boilers, and furnaces
Strengthen incinerator facilities management	NOx, SOx, PM	Waste incinerator
Restrict solvent content	volatile organic compounds (VOCs)	Solvents
Install Gas station vapor recovery system (Stage II) gradual attachment	VOCs	Fuel extraction
Apply VOC emissions standards for washing facility and printing facility	VOCs	Solvents
Establishment of special measures to reduce VOC for Gwangyang Bay area	VOCs	Industrial process
Strengthen laundry solvent management	VOCs	Solvents
Support and mandate low-NOx boiler replacement	NOx	Residential/commercial boilers
Establish VOC content standards for consumer products	VOCs	Solvents
Regulation of VOC emissions in the industry process (oil product industry, food and beverage industry)	VOC	Industrial process
Strengthen emissions standards for vehicles	NOx, PM, VOC	Light-duty vehicles (gasoline and diesel)
Supply low-emission vehicles	NOx, PM, VOC	Light-duty vehicles (gasoline and diesel)
Supply natural gas (CNG) buses and LNG vehicles	NOx, PM, VOC	Heavy-duty trucks and Bus(diesel)
Supply electric two-wheeled vehicles	NOx, VOC	Motorcycles
Strengthen emissions standards for construction machinery	NOx, SOx, PM	Construction machinery
Early retirement of old vehicles	NOx, PM, VOC	Light-duty Vehicles and Heavy-duty trucks (diesel)
Install Diesel Particulate Filter (DPF) in old vehicles	PM	Light-duty Vehicles and Heavy-duty trucks (diesel)
Replacement of three-way catalytic converter	NOx	Light-duty Vehicles and Heavy-duty trucks (gasoline)
Install PM-NOx simultaneous reduction device	NOx, PM	Heavy-duty trucks and Bus (diesel)

### Scenario C: MFR (maximum feasible reduction)

The MFR scenario envisions a gradual shift toward the implementation of the Best Available Techniques (BATs) by 2030, considering each region, sector, and fuel type. This scenario incorporates BATs, state-of-the-art emission reduction technologies available in the control strategy database of the GAINS model. A list of the technologies applied in the MFR scenario is summarized in Table 7, compiled by considering available technologies in Korea from the database. These technologies, integrated into the GAINS-Korea model, can simultaneously reduce emissions for multiple pollutants, exhibit higher emission reduction efficiency compared to existing technologies, or meet more stringent emission allowance standards^29^. The last column in Table 7 includes references^30–32^ for the BATs.

**Table 7:** Measures applied for the Maximum Feasible Reduction (MFR) scenario.

Technology	Pollutant	Sector	References
SCR	NOx	Power plant boilers (coal, oil, and gas)	^30^
High-efficiency FGD	SOx	Power plant boilers (coal, oil, and waste fuels)	^31^
Combustion modification on small biomass boilers, SCR on large boilers	NOx, SOx, PM_2.5_	Power plants (biomass)	^30–32^
Fabric filters on large boilers, good housekeeping for smaller boilers	PM_2.5_	Power plants (oil)	^32^
High-efficiency de-dusters (cyclons and fabric filters)	PM_2.5_	Commercial boilers (coal )	^32^
New boilers or stoves	PM_2.5_	Residential boilers and stoves (coal)	^32^
Catalytic inserts	PM_2.5_	Residential stoves and fireplaces (wood)	^32^
Combustion modification and low-sulfur coal and oil	NOx, SOx	Residential/commercial boilers	^30,31^
Good housekeeping	PM_2.5_	Residential/commercial boilers (oil)	^32^
SCR on larger boilers, SNCR on smaller boilers, FGD on larger boilers, in-furnace controls for smaller boilers	NOx, SOx	Industrial boilers and furnaces	^30,31^
Stage 3 controls, High-efficiency de-dusters (electrostatic precipitators or fabric filters), good practices for fugitive emissions	NOx, SOx, PM_2.5_	Industrial processes	^30–32^
Post-EURO IV (EURO VI)	NOx	Light-duty vehicles (gasoline and diesel)	^30^
Post-EURO V (EURO VI)	NOx	Heavy-duty trucks (gasoline and diesel)	^30^
Stage 3 controls	NOx	Mopeds, motorcycles	^30^
Equivalent to EURO VI on Heavy duty vehicles (post-stage III or IV, depending on a sector and rated power)	NOx	Non-road diesel vehicles (construction, agriculture, inland waterways, railways)	^30^
3-way catalytic converters	NOx	Non-road gasoline vehicles (construction, agriculture, inland waterways, railways)	^30^
Sulfur-free gasoline and diesel	SOx	Transport (land-based sources)	^31^
Low-sulfur marine oils (heavy fuel oil and diesel)	SOx	Sea transport	^31^
Good practices, feed modifications, low till farming, and alternative cereal harvesting	PM_2.5_	Agriculture	^32^
Spraying water at construction places	PM_2.5_	Construction	^32^
Good practices	PM_2.5_	Flaring in oil and gas industry	^32^

Furthermore, this scenario accounts for the lifespan of reduction facilities, which typically ranges from 20 to 30 years. It assumes a rapid adoption of BAT by 2030. The scenario focuses solely on achieving the maximum feasible reduction in emissions, without taking into consideration factors such as implementation feasibility, cost-effectiveness, or other considerations. It includes both ongoing and planned policies, applying the most efficient emission reduction technologies possible. By adopting this scenario, valuable insights can be obtained regarding the potential extent of emission reductions achievable through maximum technological efforts, as well as valuable information on the efficacy of currently planned policies and their associated impact.

## Results and discussion

**Figure 6:**
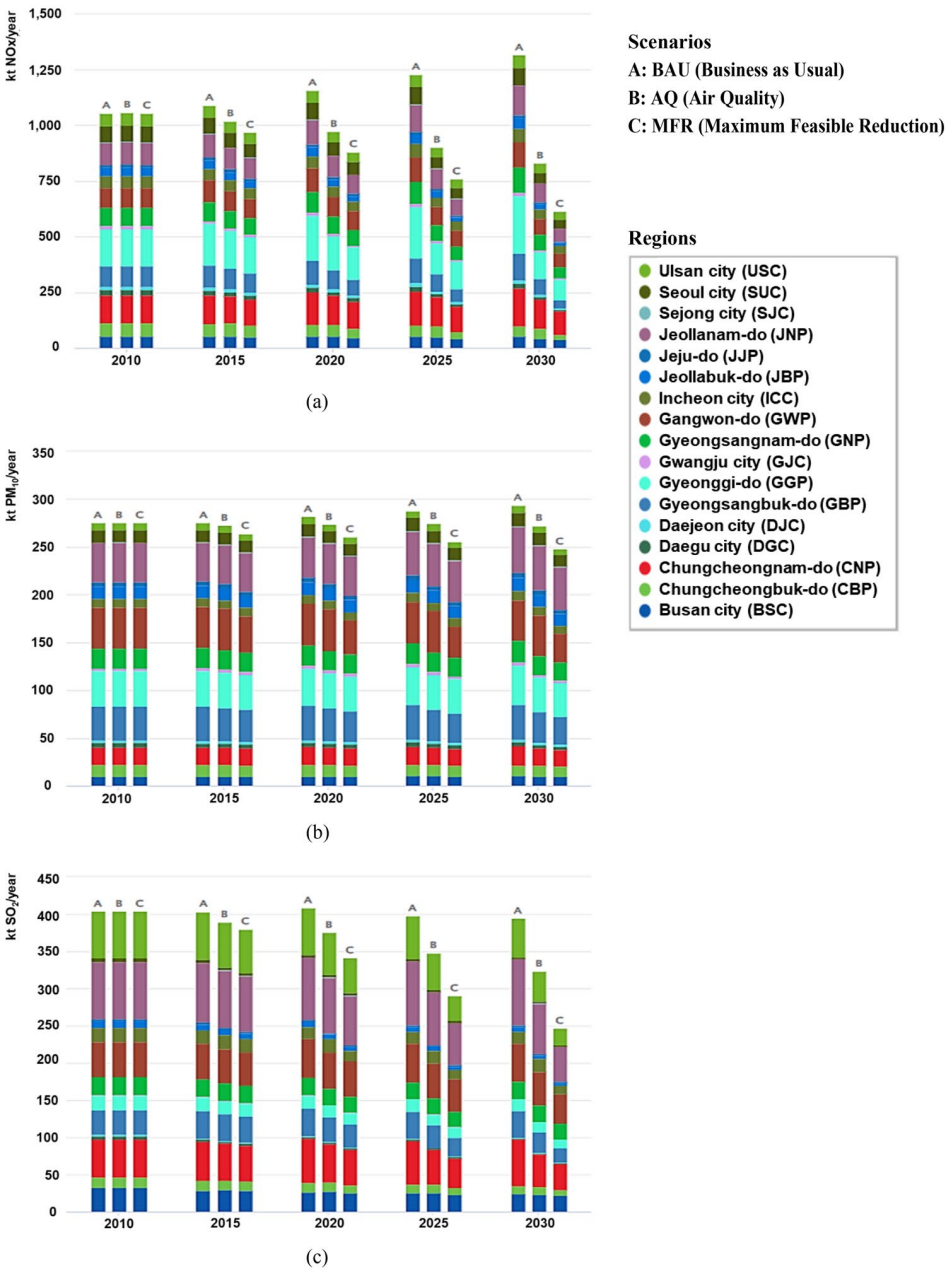
Regional Emissions by Scenarios (**a**: NOx, **b**: PM_10_, **c**: SO_2_).

**Figure 7:**
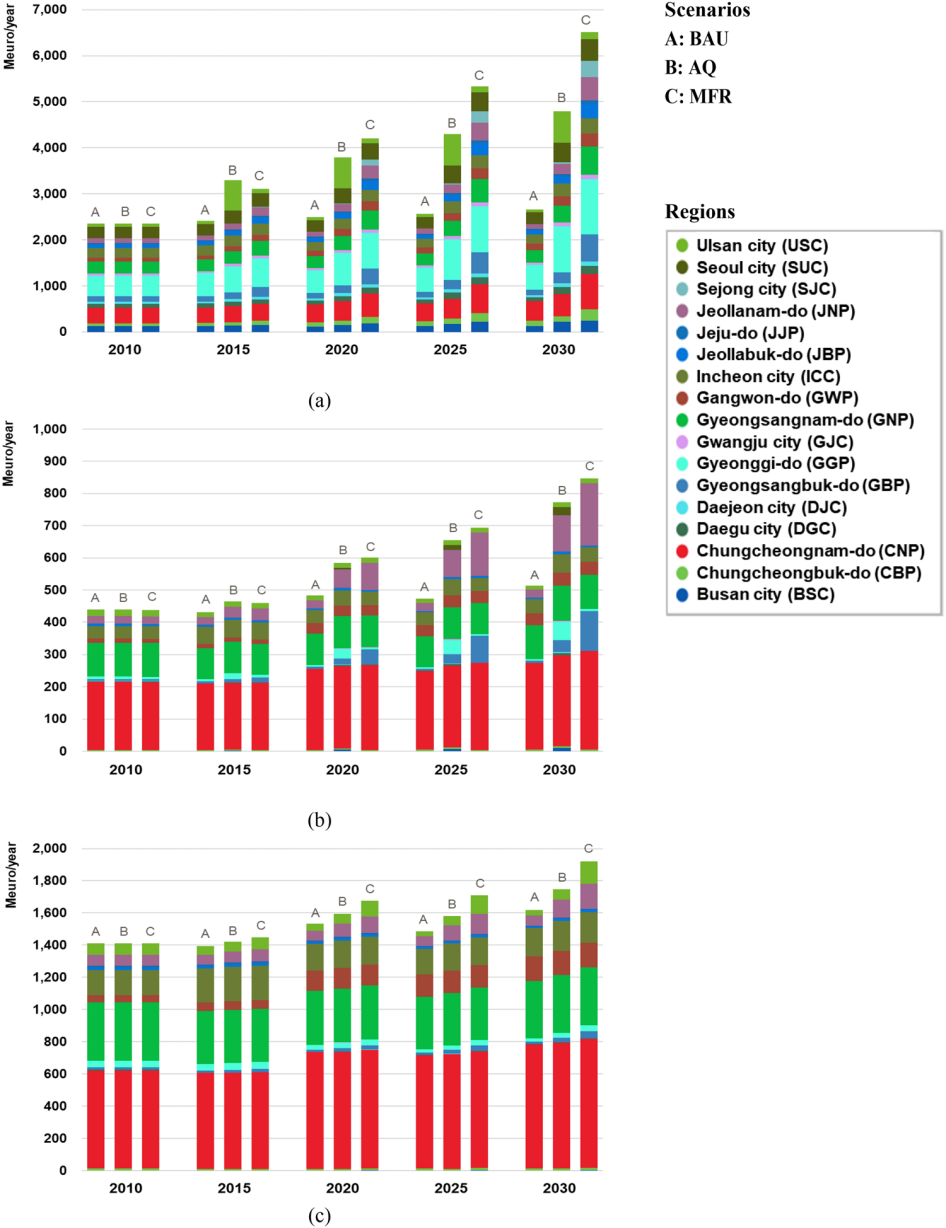
Cost of Emission Reduction by Air Pollutant (**a**: NOx, **b**: PM_10_, **c**: SO_2_).

**Figure 8:**
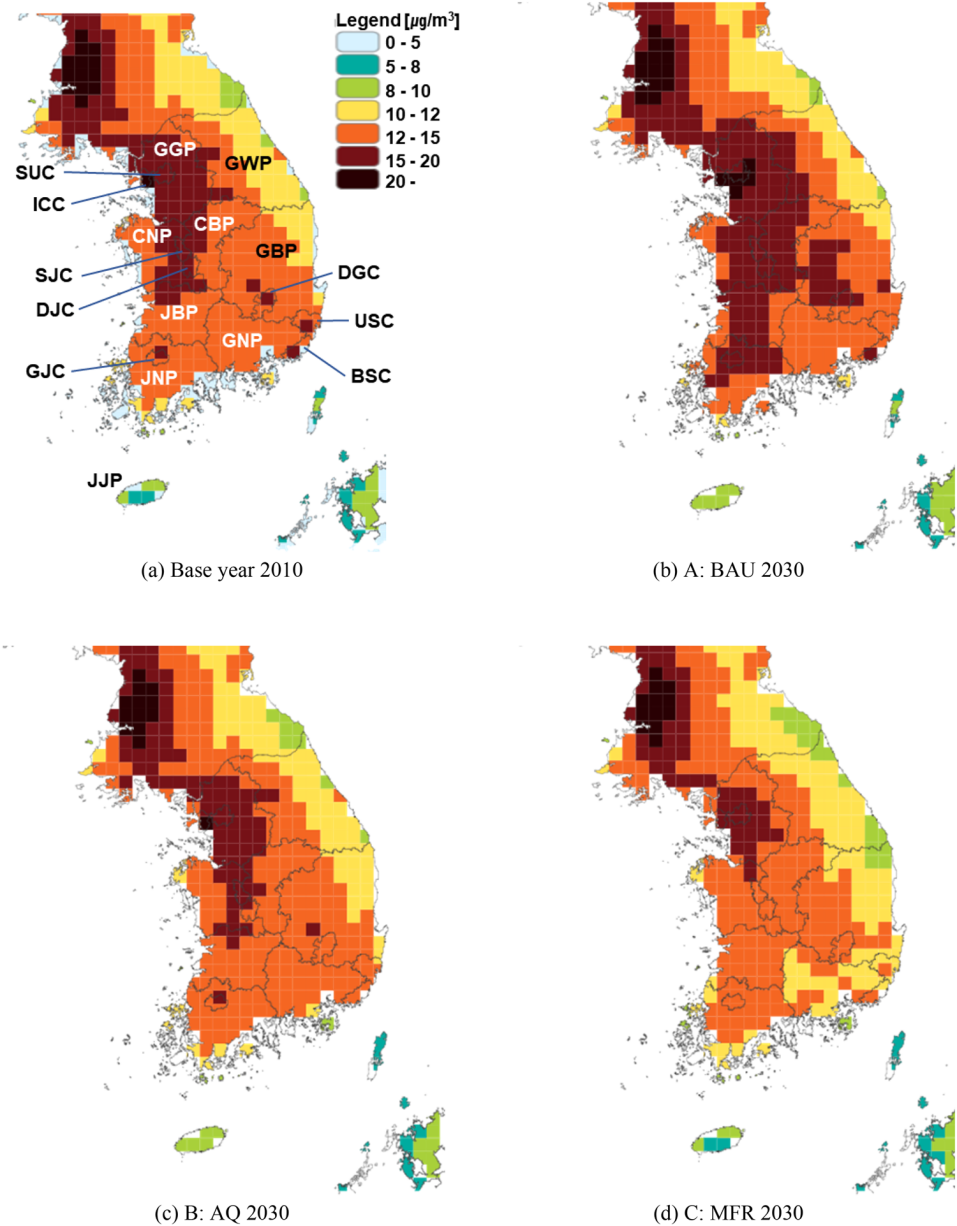
Regional PM_2.5_ concentrations by scenarios in Year 2030. These maps were generated using GAINS-Korea, which has the capability to create concentration result maps based on input scenarios. GAINS-Korea can be accessed via the web (https://gains.iiasa.ac.at/gains3/ROK/index.login?logout=1&switch_version=v0), but it necessitates a user ID and password which are managed by the International Institute for Applied Systems Analysis (IIASA).

**Figure 9:**
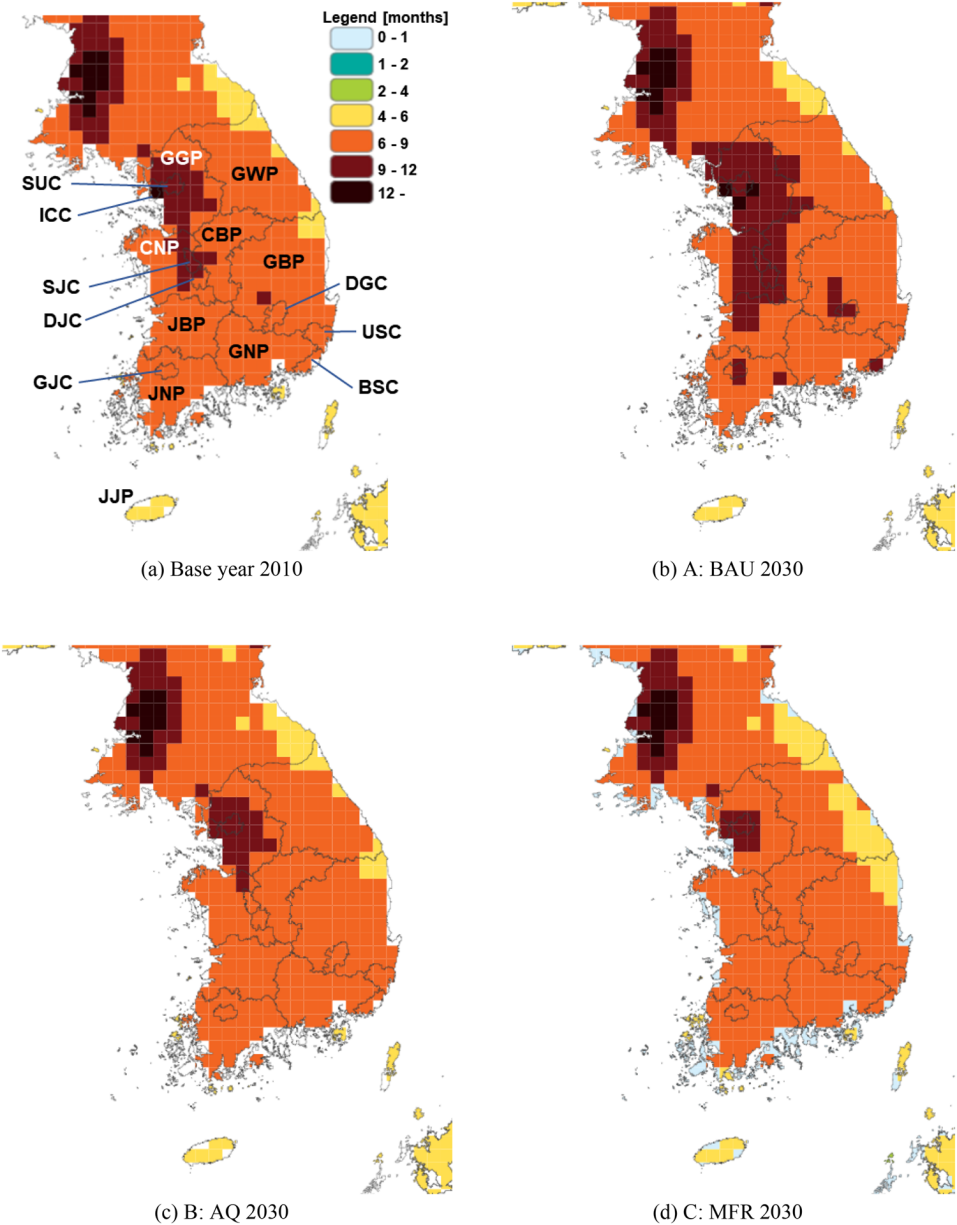
Regional Life Loss by Scenarios in Year 2030. These maps were generated using GAINS-Korea, which has the capability to create life loss result maps based on input scenarios. GAINS-Korea can be accessed via the web (https://gains.iiasa.ac.at/gains3/ROK/index.login?logout=1&switch_version=v0), but it necessitates a user ID and password which are managed by the International Institute for Applied Systems Analysis (IIASA).

The GAINS-Korea model, developed as part of this study, was utilized to analyze three distinct scenarios: BAU, AQ, and MFR. This analysis compared the emissions, PM concentrations, and human health impacts across the scenarios. Graphical representations of the analysis results obtained from GAINS-Korea for scenario comparison are presented in Figs. 6, 7, 8, and 9. The reference year is 2010, and the modeling period compares the years from 2015 to 2030, at five-year intervals. While the analysis results include various air pollutants, such as CO, NOx, SO_2_, PM, VOC, and NH_3_, as well as greenhouse gases CO_2_, N_2_O, and CH_4_, this discussion focuses on the key pollutants NOx, PM_10_, and SO_2_. The results related to these pollutants are presented and explained in detail.

### Emissions

Figure 6 depicts the projected emission trends by region at five-year intervals from 2010 to 2030 for the three scenarios. It illustrates the future changes in emissions for the key pollutants, namely NOx, PM_10_, and SO_2_. It is noteworthy that the AQ scenario exhibits a reduction in emissions for all pollutants compared to the BAU scenario, whereas the MFR scenario exhibits an even greater reduction. These findings confirm the expected results based on the extent of emission reduction policies applied. The bar graph associated with each scenario employs distinct colors to represent regional emissions, facilitating the identification of regions with higher emission levels.

The comparison between the BAU scenario and the AQ scenario revealed the changes in emissions from 2010 to 2030, allowing for an evaluation of the effectiveness of the AQ scenario, which incorporates planned policies in Korea. Analyzing the emission reduction rates for each pollutant provides insights into the effectiveness of the corresponding reduction policies. Tables S1–S3 present the estimated emissions of NOx, PM_10_, and SO_2_ by each scenario from 2010 to 2030.

- The BAU scenario projects a 25% increase in NOx emissions from 2010 to 2030, while the AQ scenario forecasts a reduction of approximately 21.2% during the same period. Compared to the BAU scenario, which assumes no additional reduction policies beyond 2030, the AQ scenario demonstrates a reduction of about 37% (1314.8–828.5 Kt). Analyzing the emission trends by sector, it becomes evident that the decrease in mobile emissions plays a significant role (refer Table S1).
- The BAU scenario predicts a 6.5% increase in PM_2.5_ emissions from 2010 to 2030, whereas the AQ scenario predicts a 1.3% reduction during the same period. Comparing the AQ scenario to the BAU scenario, the AQ scenario demonstrates a decrease of approximately 9.8%. Regarding PM_10_, which includes coarse particles, the sector associated with coarse particles contributes significantly to the total PM_10_ emissions. Since no specific reduction policies target coarse particles, the relative reduction rate in the AQ scenario is relatively lower compared to other air pollutants as we project into the future (refer Table S2)
- The comparison between the BAU and AQ scenarios reveals notable disparities in SO_2_ emissions. In the BAU scenario, there is a modest 2.5% decline in SO_2_ emissions from 2010 to 2030. Conversely, the AQ scenario exhibits a substantial reduction of approximately 19.9% over the same time frame. Consequently, the AQ scenario achieves a decrease of approximately 38.9% compared to the BAU scenario. Particularly noteworthy is the remarkable reduction rate in the domestic sector. This considerable decline in SO_2_ emissions can be attributed to the assumed reductions in coal and oil consumption activities in the residential and commercial sectors, as well as the implementation of policies promoting the use of low-sulfur fuels and the adoption of low-NOx boilers, among other measures (refer Table S3).

The analysis compared emissions across different scenarios in 2030 against those in 2020 to assess the effects of emission reduction within each scenario and region. It was evident that the policy impacts differed based on regional plans and the implemented reduction strategies. The major reduction effects by substance are outlined as follows.

- Analyzing the emissions shift in the BAU scenario for NOx revealed a national average increase of 13.8%. Across most regions, emissions in 2030 surpassed those recorded in 2020. Conversely, the AQ scenario showed a national average decrease of 14.3%, validating the efficacy of planned policies in reducing emissions. However, Chungcheongnam-do (CNP) experienced a 2% increase, presenting elevated emissions despite implemented reduction policies, driven by heightened demand for large thermal power plants. In the MFR scenario, characterized by a more robust reduction policy, the national average emissions decreased by 30.1% (refer to Table S1).
- In the BAU scenario for PM_10_, there was a national average emissions increase of 4.1%. Across most regions, emissions in 2030 surpassed those of 2020. Under the AQ scenario, the national average emissions decreased by 0.8%. However, in Gyeongsangnam-do (GNP), there was a notable 10.4% increase in emissions, primarily from increased emissions at large industrial point sources, aligning with the region's manufacturing-focused development plan. This highlights the necessity for additional reduction policies within this industrial sector. Conversely, in the MFR scenario, the national average emissions decreased by 4.6%. Particularly, Gangwon-do (GWP) showed the most substantial decrease, specifically in emissions from commercial trucks among on-road mobile emissions, demonstrating the effectiveness of the policy targeting commercial truck emissions (refer to Table S2).
- In the BAU scenario for SO_2_, the national average emissions decreased by 3.6%. Across most regions, emissions in 2030 were lower than those in 2020. However, in the AQ scenario, there was a more pronounced decline in the national average emissions, reaching 13.9%. Notably, Incheon city (ICC) was an exception, experiencing an increase in emissions attributed to the expanding power generation sector, aligned with plans to construct coal-fired power plants in the area. Under the MFR scenario, the national average emissions decreased by 27.7%. The most substantial reduction occurred in Ulsan city (USC), with emissions from power generation, industrial, and household sectors decreasing by over 50%. This emphasizes the effectiveness of strategies aimed at reducing emissions (refer to Table S3).

### Costs

Figure 7 illustrates the cost outlook by region at five-year intervals from 2010 to 2030 for three different scenarios. It shows the cost trends associated with key pollutants, namely NOx, PM_10_, and SO_2_. Tables S4–S6 include the calculated costs for reducing emissions of NOx, PM_10_, and SO_2_ by each scenario from 2010 to 2030. The AQ scenario incurs higher costs than those of the BAU scenario, and the MFR scenario incurs even higher costs than those of the AQ scenario. This observation underscores the fact that as more rigorous mitigation measures are implemented, the corresponding costs escalate. It demonstrates the direct correlation between the implementation of extensive mitigation measures and the associated financial burden from 2010 to 2030.

### PM_2.5_ concentration and health impact

In Fig. 8, a comparison of PM_2.5_ concentrations in 2030 among the three scenarios, relative to 2010 levels, is presented. It is evident that scenarios with more extensive policy measures exhibit significant improvements in PM_2.5_ concentrations. Consequently, as depicted in Fig. 9, it can be concluded that implementing more mitigation policies leads to a reduction in health impacts resulting from PM_2.5_ exposure. However, it should be noted that the concentration damage module in the GAINS-Korea model considers not only the health effects of PM_2.5_ exposure but also the population distribution when calculating the impacts. This leads to variations in the rate of concentration improvement and reductions in health impacts. This discrepancy is apparent from the lack of precise alignment between the patterns of change depicted in Figs. 8 and 9.

## Conclusions

In this study, GAINS-Korea was developed as a powerful tool specifically designed to analyze and evaluate the health impacts and costs of implementing policies aimed at enhancing air quality and mitigating greenhouse gas emissions. The emissions estimation module employed a mapping process to construct the GAINS-Korea dataset, using CAPSS and GHG-CAPSS databases, which include emission activities and emission factors. Additionally, the GAINS-Korea model incorporates policy scenarios and control technologies specific to South Korea. The CAMx, a regional-scale chemistry transport model, was used to generate source-receptor (S-R) matrices for ozone and particulate matter concentrations. These matrices elucidate the response of diverse air quality indicators to changes in pollutant emissions across different source regions. The integration of all developed modules, including the impact assessment module, within GAINS-Korea enables comprehensive air quality policy scenario analyses for South Korea.

Based on the GAINS-Korea model, several representative scenarios were devised for South Korea. The BAU scenario projected emissions until the year 2030. Additionally, two alternative scenarios were created. The AQ scenario incorporated air pollutant reduction policies aligned with the air quality control plan and the greenhouse gas reduction plan for South Korea. Conversely, the MFR scenario integrated more aggressive reduction technologies in addition to the AQ scenario. By implementing these scenarios, we were able to evaluate the impacts of emission reduction policies on the 17 local governments in South Korea, including changes in PM concentrations and the associated benefits. The results of the AQ scenarios demonstrated a notable reduction in emissions compared to the BAU scenario, indicating the effectiveness of the planned air pollution control policies. Furthermore, the MFR scenario, which assumed the application of cutting-edge technological emission reduction measures, exhibited even greater reduction effects. However, it was also analyzed that achieving such effects would require higher costs. The comparison of emissions between 2020 and 2030 across the scenarios aimed to evaluate the impact of emission reduction within specific regions and scenarios. The discernible variations in policy impacts were influenced by regional plans and the strategies implemented for reduction.

In this study, only the scenario mode was developed, and there is a need for future development of the optimization mode. This functionality of the optimization mode allows for the identification of policy combinations that minimize costs while effectively achieving targets, ensuring that reduction policies are planned to avoid exceeding future concentration limits. Although challenging, the implementation of this feature is crucial for enhancing the utility of GAINS-Korea.

It is expected that the GAINS-Korea model will play a crucial role in supporting climate and air quality research as well as informing environmental policy decisions. As with other integrated models, it is pertinent to regularly update the database with the latest data. This entails incorporating up-to-date information on emission inventories and energy consumption and enhancing the accuracy of future estimations. Furthermore, the reconstruction of the source-receptor (S-R) matrix to reflect current meteorological conditions and emission characteristics is necessary. Additionally, keeping the model updated with information on emerging emission reduction technologies is vital for effective policy evaluation.

In fact, the possibility of collecting actual data from 2010, serving as the base year of development, until the early 2020s, has become feasible, offering an opportunity to procure and integrate this historical data into the model database. This integration notably enhances the model's precision in making future predictions. However, given that the model primarily operates by predicting the future based on past data, the forecasted results for 2020–2030 in this paper hold significant value. Future studies will enable the comparison of newly collected actual data with predicted results, providing valuable insights for evaluating and refining the performance of the GAINS-Korea model.

Furthermore, in Korea, the necessity to forecast future emissions, particularly targeting 2050, has spurred ongoing research efforts. These endeavors involve updating historical data, gathering information on future reduction policies, and seamlessly integrating these components into the model. The outcomes of these efforts will be integrated into the forthcoming updated version. Continuous support and ongoing improvement endeavors remain pivotal in strengthening GAINS-Korea, ensuring its sustained relevance and utility for policymakers.

### Supplementary Information

Supplementary Information.

## Data Availability

The authors confirm that the data supporting the findings of this study are available within the article [and/or] its supplementary materials. If needed, the data utilized in this study can be obtained from the corresponding author (H.H.) upon a reasonable request.
