# Supplementary material for: Development of a greenhouse gas - air pollution interactions and synergies model for Korea (GAINS-Korea)
Source: Sci Rep. 2024 Feb 9;14:3372. doi: 10.1038/s41598-024-53632-w (PMC10858138; doi:10.1038/s41598-024-53632-w)
Supplement: Supplementary file 1 — Supplementary Information. [file 41598_2024_53632_MOESM1_ESM.docx]

| Region | yr2010 | | | yr2015 | | | yr2020 | | | yr2025 | | | yr2030 | | | **((yr2030-yr2010)/yr2010)**  ***100** | | | | **((yr2030-yr2020)/yr2020)**  ***100** | | | |
| --- | --- | --- | --- | --- | --- | --- | --- | --- | --- | --- | --- | --- | --- | --- | --- | --- | --- | --- | --- | --- | --- | --- | --- |
| scenarios | BAU | AQ | MFR | BAU | AQ | MFR | BAU | AQ | MFR | BAU | AQ | MFR | BAU | AQ | MFR | BAU | AQ | MFR | BAU | | AQ | MFR |  |
| Busan city (BSC) | 53.6 | 53.6 | 53.6 | 52.8 | 54.6 | 49.4 | 52.8 | 51.5 | 45.5 | 52.9 | 49.7 | 41.6 | 52.8 | 41.9 | 37.6 | -1.4% | -21.7% | -29.8% | 0.0% | | -18.5% | -17.3% |  |
| Chungcheongbuk-do (CBP) | 60.8 | 60.8 | 60.8 | 57.8 | 57.8 | 52.0 | 54.9 | 54.9 | 43.1 | 51.3 | 51.3 | 33.2 | 46.1 | 46.1 | 20.9 | -24.1% | -24.1% | -65.6% | -15.9% | | -15.9% | -51.4% |  |
| Chungcheongnam-do (CNP) | 123.8 | 123.8 | 123.8 | 125.5 | 120.0 | 117.2 | 143.0 | 129.8 | 121.3 | 151.8 | 128.5 | 112.5 | 170.1 | 132.4 | 107.4 | 37.4% | 6.9% | -13.3% | 18.9% | | 2.0% | -11.5% |  |
| Daegu city (DGC) | 23.7 | 23.7 | 23.7 | 22.3 | 19.6 | 19.1 | 22.1 | 16.9 | 15.6 | 21.9 | 14.1 | 12.0 | 21.4 | 11.3 | 8.3 | -9.4% | -52.4% | -64.8% | -3.1% | | -33.3% | -46.5% |  |
| Daejeon city (DJC) | 14.3 | 14.3 | 14.3 | 14.0 | 12.8 | 12.2 | 14.7 | 11.9 | 10.5 | 15.4 | 10.9 | 8.5 | 15.8 | 9.6 | 6.2 | 11.1% | -32.9% | -56.5% | 8.0% | | -19.7% | -41.0% |  |
| Gangwon-do (GWP) | 89.7 | 89.7 | 89.7 | 97.1 | 87.2 | 85.3 | 108.9 | 88.0 | 83.9 | 111.4 | 79.2 | 72.5 | 114.1 | 69.7 | 59.7 | 27.2% | -22.3% | -33.4% | 4.7% | | -20.8% | -28.8% |  |
| Gwangju city (GJC) | 11.4 | 11.4 | 11.4 | 11.8 | 10.6 | 10.1 | 12.3 | 9.8 | 8.7 | 12.8 | 8.7 | 7.0 | 13.1 | 7.5 | 5.1 | 14.6% | -34.8% | -55.5% | 6.4% | | -23.9% | -41.5% |  |
| Gyeonggi-do (GGP) | 166.2 | 166.2 | 166.2 | 187.6 | 171.0 | 162.6 | 203.8 | 153.8 | 143.3 | 231.4 | 138.0 | 124.1 | 261.5 | 122.4 | 91.9 | 57.4% | -26.4% | -44.7% | 28.3% | | -20.5% | -35.9% |  |
| Gyeongsangbuk-do (GBP) | 94.4 | 94.4 | 94.4 | 100.7 | 93.0 | 88.0 | 105.7 | 86.5 | 73.4 | 111.7 | 79.6 | 57.1 | 118.2 | 70.3 | 37.3 | 25.2% | -25.5% | -60.5% | 11.8% | | -18.8% | -49.2% |  |
| Gyeongsangnam-do (GNP) | 83.0 | 83.0 | 83.0 | 84.5 | 78.0 | 74.6 | 91.3 | 77.8 | 70.2 | 99.1 | 74.1 | 61.5 | 111.7 | 69.9 | 50.7 | 34.7% | -15.8% | -38.9% | 22.4% | | -10.1% | -27.9% |  |
| Incheon city (ICC) | 51.7 | 51.7 | 51.7 | 52.5 | 50.0 | 47.8 | 53.7 | 45.6 | 42.9 | 57.2 | 42.9 | 39.4 | 61.4 | 41.5 | 35.5 | 18.8% | -19.7% | -31.3% | 14.4% | | -9.0% | -17.2% |  |
| Jeju-do (JJP) | 10.8 | 10.8 | 10.8 | 11.4 | 14.2 | 10.5 | 11.3 | 12.0 | 8.6 | 11.4 | 9.9 | 7.0 | 12.2 | 9.6 | 5.6 | 12.9% | -11.3% | -48.1% | 8.2% | | -20.0% | -34.7% |  |
| Jeollabuk-do (JBP) | 40.2 | 40.2 | 40.2 | 38.7 | 35.0 | 32.7 | 41.3 | 32.5 | 27.4 | 44.7 | 29.5 | 21.0 | 49.0 | 25.5 | 13.1 | 21.9% | -36.5% | -67.4% | 18.7% | | -21.5% | -52.1% |  |
| Jeollanam-do (JNP) | 98.7 | 98.7 | 98.7 | 103.8 | 96.5 | 93.5 | 110.6 | 92.9 | 84.5 | 120.7 | 89.0 | 74.2 | 131.7 | 82.4 | 60.4 | 33.5% | -16.4% | -38.7% | 19.1% | | -11.3% | -28.5% |  |
| Sejong city (SJC) | 2.5 | 2.5 | 2.5 | 2.3 | 2.0 | 1.4 | 2.4 | 1.6 | 1.0 | 2.5 | 1.3 | 0.7 | 2.6 | 0.9 | 0.3 | 4.5% | -65.5% | -87.5% | 8.4% | | -47.6% | -70.0% |  |
| Seoul city (SUC) | 70.7 | 70.7 | 70.7 | 71.3 | 65.9 | 62.8 | 74.1 | 58.8 | 55.2 | 76.8 | 51.5 | 46.9 | 78.6 | 46.1 | 37.6 | 11.2% | -34.8% | -46.8% | 6.0% | | -21.6% | -31.9% |  |
| Ulsan city (USC) | 56.8 | 56.8 | 56.8 | 54.3 | 51.1 | 50.3 | 52.8 | 46.6 | 44.5 | 53.6 | 44.1 | 40.9 | 54.3 | 41.6 | 37.2 | -4.4% | -26.9% | -34.6% | 2.8% | | -10.9% | -16.5% |  |
| **Total** | **1,052.1** | **1,052.1** | **1,052.1** | **1,088.4** | **1,019.3** | **969.6** | **1,155.7** | **970.9** | **879.7** | **1,226.6** | **902.4** | **760.1** | **1,314.8** | **828.5** | **614.9** | **25.0%** | **-21.2%** | **-41.5%** | **13.8%** | | **-14.7%** | **-30.1%** |  |

**Supplementary Table S1.** Estimated emissions of NOx by each scenario from 2010 to 2030. (unit: Kt)

| Region | yr2010 | | | yr2015 | | | yr2020 | | | yr2025 | | | yr2030 | | | **((yr2030-yr2010)/yr2010)**  ***100** | | | | **((yr2030-yr2020)/yr2020)**  ***100** | | | |
| --- | --- | --- | --- | --- | --- | --- | --- | --- | --- | --- | --- | --- | --- | --- | --- | --- | --- | --- | --- | --- | --- | --- | --- |
| scenarios | BAU | AQ | MFR | BAU | AQ | MFR | BAU | AQ | MFR | BAU | AQ | MFR | BAU | AQ | MFR | BAU | AQ | MFR | BAU | | AQ | MFR |  |
| Busan city (BSC) | 9.9 | 9.9 | 9.9 | 9.8 | 10.1 | 9.7 | 10.2 | 10.3 | 9.8 | 10.4 | 10.4 | 9.9 | 10.5 | 9.9 | 9.8 | 6.7% | 0.6% | -0.5% | 3.7% | | -3.3% | 0.2% |  |
| Chungcheongbuk-do (CBP) | 12.8 | 12.8 | 12.8 | 12.2 | 12.2 | 12.1 | 12.0 | 12.0 | 11.8 | 11.7 | 11.7 | 11.5 | 11.3 | 11.3 | 10.9 | -11.4% | -11.4% | -14.6% | -5.7% | | -5.7% | -7.9% |  |
| Chungcheongnam-do (CNP) | 18.2 | 18.2 | 18.2 | 18.4 | 18.0 | 18.0 | 19.1 | 18.3 | 17.9 | 19.6 | 18.2 | 17.5 | 20.3 | 18.1 | 17.0 | 11.7% | -0.5% | -6.6% | 6.1% | | -1.4% | -5.4% |  |
| Daegu city (DGC) | 4.5 | 4.5 | 4.5 | 4.4 | 4.2 | 4.2 | 4.3 | 4.1 | 4.1 | 4.3 | 4.0 | 4.0 | 4.3 | 3.9 | 3.8 | -3.5% | -12.5% | -13.8% | -0.9% | | -5.6% | -6.5% |  |
| Daejeon city (DJC) | 2.6 | 2.6 | 2.6 | 2.5 | 2.5 | 2.5 | 2.6 | 2.5 | 2.4 | 2.6 | 2.4 | 2.4 | 2.7 | 2.4 | 2.3 | 4.7% | -6.5% | -8.9% | 2.9% | | -3.2% | -4.7% |  |
| Gangwon-do (GWP) | 42.9 | 42.9 | 42.9 | 43.2 | 43.4 | 38.1 | 43.5 | 43.9 | 35.7 | 43.2 | 43.7 | 32.8 | 42.5 | 42.6 | 29.6 | -1.0% | -0.8% | -31.1% | -2.2% | | -2.9% | -17.0% |  |
| Gwangju city (GJC) | 2.8 | 2.8 | 2.8 | 2.8 | 2.8 | 2.8 | 2.9 | 2.8 | 2.8 | 3.0 | 2.8 | 2.8 | 3.0 | 2.8 | 2.8 | 7.1% | -0.2% | -2.5% | 3.6% | | -0.3% | -1.5% |  |
| Gyeonggi-do (GGP) | 37.2 | 37.2 | 37.2 | 37.7 | 37.0 | 36.8 | 38.8 | 36.9 | 36.6 | 40.0 | 36.4 | 36.2 | 41.2 | 35.9 | 35.2 | 10.9% | -3.5% | -5.5% | 6.1% | | -2.7% | -4.0% |  |
| Gyeongsangbuk-do (GBP) | 35.5 | 35.5 | 35.5 | 35.9 | 35.2 | 33.7 | 36.3 | 34.5 | 32.5 | 36.4 | 33.6 | 31.0 | 36.4 | 32.5 | 29.2 | 2.7% | -8.5% | -17.5% | 0.5% | | -5.9% | -9.9% |  |
| Gyeongsangnam-do (GNP) | 20.7 | 20.7 | 20.7 | 20.8 | 20.4 | 20.2 | 21.3 | 20.3 | 19.9 | 21.7 | 20.1 | 19.4 | 22.4 | 19.7 | 18.7 | 8.0% | -5.1% | -9.4% | 5.2% | | -3.2% | -5.8% |  |
| Incheon city (ICC) | 8.9 | 8.9 | 8.9 | 8.9 | 8.8 | 8.7 | 9.2 | 8.9 | 8.8 | 9.5 | 8.9 | 8.9 | 9.8 | 8.9 | 8.8 | 10.3% | 0.6% | -0.9% | 6.3% | | 0.8% | -0.3% |  |
| Jeju-do (JJP) | 4.3 | 4.3 | 4.3 | 4.3 | 4.4 | 4.2 | 4.5 | 4.5 | 4.4 | 4.6 | 4.6 | 4.5 | 4.7 | 4.6 | 4.5 | 10.1% | 8.9% | 5.5% | 4.8% | | 3.1% | 2.4% |  |
| Jeollabuk-do (JBP) | 13.2 | 13.2 | 13.2 | 13.2 | 12.9 | 12.8 | 13.4 | 12.9 | 12.7 | 13.6 | 12.8 | 12.4 | 13.9 | 12.6 | 12.1 | 5.1% | -4.7% | -8.7% | 3.6% | | -2.5% | -4.8% |  |
| Jeollanam-do (JNP) | 41.3 | 41.3 | 41.3 | 40.7 | 40.3 | 40.1 | 42.9 | 42.0 | 41.3 | 45.9 | 44.3 | 43.1 | 48.8 | 46.3 | 44.6 | 18.0% | 12.1% | 7.8% | 13.7% | | 10.4% | 8.0% |  |
| Sejong city (SJC) | 0.5 | 0.5 | 0.5 | 0.6 | 0.6 | 0.6 | 0.7 | 0.7 | 0.7 | 0.7 | 0.7 | 0.6 | 0.7 | 0.6 | 0.6 | 44.3% | 30.5% | 24.6% | -1.5% | | -7.0% | -7.2% |  |
| Seoul city (SUC) | 12.8 | 12.8 | 12.8 | 12.8 | 12.7 | 12.6 | 13.3 | 12.9 | 12.9 | 13.6 | 13.0 | 13.0 | 13.8 | 13.0 | 13.0 | 7.8% | 1.6% | 1.1% | 3.9% | | 0.9% | 0.6% |  |
| Ulsan city (USC) | 7.5 | 7.5 | 7.5 | 7.1 | 7.0 | 6.5 | 7.0 | 6.8 | 6.1 | 7.1 | 6.8 | 5.8 | 7.2 | 6.8 | 5.5 | -4.6% | -10.3% | -27.0% | 2.3% | | -0.9% | -9.7% |  |
| **Total** | **275.5** | **275.5** | **275.5** | **275.3** | **272.6** | **263.7** | **282.0** | **274.3** | **260.4** | **288.2** | **274.4** | **255.5** | **293.5** | **272.0** | **248.3** | **6.5%** | **-1.3%** | **-9.8%** | **4.1%** | | **-0.8%** | **-4.6%** |  |

**Supplementary Table S2.** Estimated emissions of PM_10_ by each scenario from 2010 to 2030. (unit: Kt)

| Region | yr2010 | | | yr2015 | | | yr2020 | | | yr2025 | | | yr2030 | | | **((yr2030-yr2010)/yr2010)**  ***100** | | | **((yr2030-yr2020)/yr2020)**  ***100** | | |
| --- | --- | --- | --- | --- | --- | --- | --- | --- | --- | --- | --- | --- | --- | --- | --- | --- | --- | --- | --- | --- | --- |
| scenarios | BAU | AQ | MFR | BAU | AQ | MFR | BAU | AQ | MFR | BAU | AQ | MFR | BAU | AQ | MFR | BAU | AQ | MFR | BAU | AQ | MFR |
| Busan city (BSC) | 33.1 | 33.1 | 33.1 | 28.7 | 29.7 | 28.4 | 26.6 | 27.4 | 25.7 | 25.2 | 25.9 | 23.8 | 24.1 | 23.2 | 22.3 | -27.1% | -29.9% | -32.5% | -9.4% | -15.4% | -13.1% |
| Chungcheongbuk-do (CBP) | 14.1 | 14.1 | 14.1 | 13.4 | 13.4 | 12.6 | 12.5 | 12.5 | 10.9 | 11.6 | 11.6 | 9.2 | 10.6 | 10.6 | 7.6 | -24.8% | -24.8% | -46.5% | -15.4% | -15.4% | -30.8% |
| Chungcheongnam-do (CNP) | 50.6 | 50.6 | 50.6 | 52.7 | 49.2 | 48.4 | 59.9 | 51.3 | 47.7 | 59.3 | 46.3 | 39.8 | 62.7 | 43.8 | 34.6 | 23.9% | -13.5% | -31.6% | 4.6% | -14.7% | -27.4% |
| Daegu city (DGC) | 4.4 | 4.4 | 4.4 | 2.7 | 2.7 | 2.7 | 2.3 | 2.3 | 2.2 | 1.9 | 1.9 | 1.8 | 1.6 | 1.6 | 1.5 | -63.3% | -64.8% | -66.1% | -29.8% | -31.5% | -33.2% |
| Daejeon city (DJC) | 1.9 | 1.9 | 1.9 | 1.2 | 1.2 | 1.2 | 1.1 | 1.0 | 1.0 | 1.0 | 0.9 | 0.9 | 0.9 | 0.7 | 0.6 | -51.4% | -64.5% | -65.7% | -19.7% | -36.6% | -38.2% |
| Gangwon-do (GWP) | 46.0 | 46.0 | 46.0 | 47.2 | 45.8 | 45.4 | 52.1 | 49.0 | 46.9 | 51.7 | 47.2 | 43.7 | 50.9 | 45.0 | 39.8 | 10.5% | -2.3% | -13.5% | -2.3% | -8.2% | -15.0% |
| Gwangju city (GJC) | 1.0 | 1.0 | 1.0 | 0.9 | 0.9 | 0.9 | 0.8 | 0.8 | 0.8 | 0.6 | 0.6 | 0.6 | 0.5 | 0.5 | 0.5 | -48.0% | -49.2% | -51.4% | -32.5% | -33.5% | -35.5% |
| Gyeonggi-do (GGP) | 18.9 | 18.9 | 18.9 | 18.9 | 18.0 | 17.8 | 17.7 | 15.9 | 15.4 | 16.7 | 14.1 | 13.3 | 16.3 | 13.1 | 11.6 | -14.1% | -30.8% | -38.6% | -8.3% | -17.3% | -24.6% |
| Gyeongsangbuk-do (GBP) | 33.7 | 33.7 | 33.7 | 36.9 | 35.2 | 34.9 | 36.4 | 32.7 | 30.0 | 36.2 | 30.5 | 25.0 | 35.8 | 28.1 | 19.4 | 6.3% | -16.6% | -42.5% | -1.6% | -14.1% | -35.5% |
| Gyeongsangnam-do (GNP) | 24.8 | 24.8 | 24.8 | 23.5 | 23.2 | 23.1 | 23.1 | 22.5 | 22.1 | 22.4 | 21.4 | 20.8 | 23.0 | 21.7 | 20.9 | -7.1% | -12.2% | -15.7% | -0.5% | -3.4% | -5.5% |
| Incheon city (ICC) | 19.4 | 19.4 | 19.4 | 18.7 | 19.1 | 17.7 | 16.4 | 17.1 | 14.2 | 15.8 | 16.9 | 12.5 | 16.1 | 17.7 | 11.6 | -17.0% | -8.5% | -40.0% | -2.0% | 3.9% | -18.1% |
| Jeju-do (JJP) | 2.3 | 2.3 | 2.3 | 2.4 | 2.3 | 2.3 | 2.1 | 1.9 | 1.9 | 1.8 | 1.6 | 1.6 | 1.8 | 1.6 | 1.6 | -22.8% | -32.2% | -32.4% | -13.5% | -16.0% | -16.2% |
| Jeollabuk-do (JBP) | 10.0 | 10.0 | 10.0 | 7.7 | 7.3 | 7.2 | 7.3 | 6.5 | 5.9 | 6.9 | 5.8 | 4.6 | 6.5 | 5.1 | 3.4 | -35.0% | -49.2% | -65.8% | -10.9% | -22.3% | -41.7% |
| Jeollanam-do (JNP) | 76.0 | 76.0 | 76.0 | 80.2 | 76.8 | 75.1 | 83.6 | 74.4 | 66.3 | 86.3 | 71.1 | 56.6 | 89.1 | 67.6 | 46.7 | 17.3% | -11.0% | -38.5% | 6.6% | -9.1% | -29.5% |
| Sejong city (SJC) | 0.1 | 0.1 | 0.1 | 0.2 | 0.2 | 0.2 | 0.3 | 0.3 | 0.3 | 0.3 | 0.3 | 0.2 | 0.3 | 0.2 | 0.2 | 184.3% | 175.3% | 137.1% | -8.0% | -9.9% | -18.8% |
| Seoul city (SUC) | 5.0 | 5.0 | 5.0 | 3.7 | 3.7 | 3.7 | 3.1 | 3.1 | 3.1 | 2.6 | 2.6 | 2.6 | 2.2 | 2.2 | 2.2 | -55.7% | -55.7% | -55.7% | -30.0% | -30.0% | -30.0% |
| Ulsan city (USC) | 63.0 | 63.0 | 63.0 | 63.6 | 61.0 | 58.8 | 63.4 | 57.3 | 47.4 | 57.5 | 48.9 | 33.5 | 51.9 | 41.2 | 22.4 | -17.6% | -34.7% | -64.5% | -18.1% | -28.1% | -52.8% |
| **Total** | **404.3** | **404.3** | **404.3** | **402.9** | **389.7** | **380.2** | **408.8** | **376.0** | **341.8** | **397.8** | **347.6** | **290.7** | **394.3** | **323.8** | **247.0** | **-2.5%** | **-19.9%** | **-38.9%** | **-3.6%** | **-13.9%** | **-27.7%** |

**Supplementary Table S3.** Estimated emissions of SO_2_ by each scenario from 2010 to 2030. (unit: Kt)

| Region | yr2010 | | | yr2015 | | | yr2020 | | | yr2025 | | | yr2030 | | | **((yr2030-yr2010)/yr2010)**  ***100** | | | | **((yr2030-yr2020)/yr2020)**  ***100** | | | |
| --- | --- | --- | --- | --- | --- | --- | --- | --- | --- | --- | --- | --- | --- | --- | --- | --- | --- | --- | --- | --- | --- | --- | --- |
| scenarios | BAU | AQ | MFR | BAU | AQ | MFR | BAU | AQ | MFR | BAU | AQ | MFR | BAU | AQ | MFR | BAU | AQ | MFR | BAU | | AQ | MFR |  |
| Busan city (BSC) | 128.0 | 128.0 | 128.0 | 123.1 | 133.7 | 151.4 | 121.2 | 156.6 | 181.4 | 124.5 | 179.3 | 216.6 | 125.7 | 216.4 | 249.5 | -1.9% | 69.0% | 94.8% | 3.7% | | 38.2% | 37.5% |  |
| Chungcheongbuk-do (CBP) | 64.1 | 64.1 | 64.1 | 77.4 | 77.4 | 91.6 | 92.2 | 92.2 | 142.8 | 108.1 | 108.1 | 195.2 | 125.4 | 125.4 | 249.3 | 95.6% | 95.6% | 288.8% | 36.1% | | 36.1% | 74.6% |  |
| Chungcheongnam-do (CNP) | 337.0 | 337.0 | 337.0 | 333.0 | 348.1 | 364.2 | 381.7 | 415.8 | 511.7 | 385.3 | 435.0 | 620.1 | 416.4 | 484.4 | 757.9 | 23.6% | 43.8% | 124.9% | 9.1% | | 16.5% | 48.1% |  |
| Daegu city (DGC) | 81.8 | 81.8 | 81.8 | 83.3 | 99.8 | 103.1 | 83.3 | 115.1 | 126.3 | 84.2 | 131.3 | 150.5 | 84.4 | 146.8 | 173.8 | 3.2% | 79.4% | 112.4% | 1.4% | | 27.6% | 37.6% |  |
| Daejeon city (DJC) | 46.7 | 46.7 | 46.7 | 46.8 | 54.5 | 57.9 | 47.0 | 63.1 | 71.3 | 47.2 | 72.1 | 85.3 | 47.3 | 80.9 | 99.0 | 1.4% | 73.2% | 112.0% | 0.7% | | 28.1% | 38.8% |  |
| Gangwon-do (GWP) | 113.5 | 113.5 | 113.5 | 117.2 | 140.9 | 212.6 | 117.9 | 172.4 | 337.5 | 119.9 | 204.4 | 464.0 | 121.1 | 236.9 | 591.9 | 6.7% | 108.7% | 421.4% | 2.7% | | 37.4% | 75.4% |  |
| Gwangju city (GJC) | 447.8 | 447.8 | 447.8 | 495.9 | 574.1 | 616.6 | 498.5 | 702.8 | 782.7 | 528.1 | 878.9 | 995.9 | 542.7 | 1001.5 | 1197.3 | 21.2% | 123.7% | 167.4% | 8.9% | | 42.5% | 53.0% |  |
| Gyeonggi-do (GGP) | 45.9 | 45.9 | 45.9 | 46.0 | 54.0 | 58.7 | 46.2 | 62.8 | 72.4 | 46.3 | 71.6 | 86.1 | 46.4 | 80.4 | 99.7 | 1.1% | 75.3% | 117.5% | 0.4% | | 28.0% | 37.9% |  |
| Gyeongsangbuk-do (GBP) | 260.6 | 260.6 | 260.6 | 250.4 | 275.0 | 323.5 | 259.0 | 305.7 | 414.3 | 259.2 | 332.3 | 501.2 | 273.0 | 375.5 | 605.1 | 4.7% | 44.1% | 132.1% | 5.4% | | 22.8% | 46.1% |  |
| Gyeongsangnam-do (GNP) | 87.3 | 87.3 | 87.3 | 90.3 | 102.7 | 124.4 | 119.3 | 145.8 | 193.6 | 127.6 | 168.5 | 242.6 | 134.1 | 189.8 | 290.3 | 53.6% | 117.4% | 232.5% | 12.5% | | 30.2% | 49.9% |  |
| Incheon city (ICC) | 211.9 | 211.9 | 211.9 | 219.3 | 233.1 | 246.2 | 192.5 | 230.4 | 249.1 | 194.2 | 256.3 | 280.8 | 204.9 | 284.6 | 321.8 | -3.3% | 34.4% | 51.9% | 6.4% | | 23.6% | 29.2% |  |
| Jeju-do (JJP) | 82.5 | 82.5 | 82.5 | 83.6 | 98.7 | 133.5 | 84.2 | 116.2 | 194.9 | 85.1 | 134.6 | 257.4 | 86.0 | 153.5 | 320.5 | 4.2% | 86.0% | 288.3% | 2.1% | | 32.1% | 64.4% |  |
| Jeollabuk-do (JBP) | 29.0 | 29.0 | 29.0 | 28.7 | 32.9 | 38.6 | 27.1 | 36.8 | 48.6 | 25.7 | 40.9 | 58.6 | 25.9 | 46.6 | 70.5 | -10.5% | 61.1% | 143.6% | -4.2% | | 26.7% | 45.1% |  |
| Jeollanam-do (JNP) | 103.7 | 103.7 | 103.7 | 104.0 | 127.2 | 181.7 | 106.9 | 157.3 | 286.9 | 107.8 | 188.3 | 393.5 | 109.8 | 222.4 | 503.5 | 5.9% | 114.4% | 385.4% | 2.8% | | 41.4% | 75.5% |  |
| Sejong city (SJC) | 0.8 | 0.8 | 0.8 | 0.8 | 7.6 | 19.6 | 0.8 | 17.9 | 132.3 | 0.8 | 28.3 | 245.0 | 0.8 | 38.6 | 357.8 | 1.3% | 4786.1% | 45188.6% | 0.0% | | 115.4% | 170.4% |  |
| Seoul city (SUC) | 242.9 | 242.9 | 242.9 | 246.8 | 277.8 | 295.9 | 249.7 | 331.2 | 352.7 | 254.8 | 386.9 | 412.1 | 256.3 | 423.2 | 467.9 | 5.5% | 74.3% | 92.7% | 2.6% | | 27.8% | 32.7% |  |
| Ulsan city (USC) | 70.1 | 70.1 | 70.1 | 67.1 | 656.3 | 87.6 | 63.5 | 664.2 | 109.4 | 64.4 | 676.5 | 133.0 | 65.4 | 689.1 | 156.1 | -6.7% | 882.9% | 122.6% | 3.0% | | 3.8% | 42.7% |  |
| **Total** | **2,353.6** | **2,353.6** | **2,353.6** | **2,413.4** | **3,293.7** | **3,107.1** | **2,490.7** | **3,786.1** | **4207.8** | **2563.0** | **4293.3** | **5,337.9** | **2,665.6** | **4,796.0** | **6,511.8** | **13.3%** | **103.8%** | **176.7%** | **7.0%** | | **26.7%** | **54.8%** |  |

**Supplementary Table S4.** Estimated costs for reducing NOx emissions by each scenario from 2010 to 2030. (unit: MEuro/year)

| Region | yr2010 | | | yr2015 | | | yr2020 | | | yr2025 | | | yr2030 | | | **((yr2030-yr2010)/yr2010)**  ***100** | | | | **((yr2030-yr2020)/yr2020)**  ***100** | | | |
| --- | --- | --- | --- | --- | --- | --- | --- | --- | --- | --- | --- | --- | --- | --- | --- | --- | --- | --- | --- | --- | --- | --- | --- |
| scenarios | BAU | AQ | MFR | BAU | AQ | MFR | BAU | AQ | MFR | BAU | AQ | MFR | BAU | AQ | MFR | **BAU** | **AQ** | **MFR** | **BAU** | | **AQ** | **MFR** |  |
| Busan city (BSC) | 0.3 | 0.3 | 0.3 | 0.3 | 1.5 | 0.4 | 0.3 | 4.5 | 0.5 | 0.3 | 7.4 | 0.7 | 0.3 | 10.5 | 0.8 | -21.2% | 3,078.8% | 148.5% | -7.1% | | 131.6% | 67.3% |  |
| Chungcheongbuk-do (CBP) | 3.0 | 3.0 | 3.0 | 2.9 | 2.9 | 2.6 | 3.8 | 3.8 | 3.1 | 4.8 | 4.8 | 3.6 | 5.6 | 5.6 | 4.0 | 88.9% | 88.9% | 34.6% | 47.0% | | 47.0% | 30.2% |  |
| Chungcheongnam-do (CNP) | 211.5 | 211.5 | 211.5 | 206.8 | 208.4 | 210.0 | 251.5 | 256.5 | 264.9 | 244.9 | 253.8 | 270.2 | 268.6 | 281.6 | 306.7 | 27.0% | 33.1% | 45.0% | 6.8% | | 9.8% | 15.8% |  |
| Daegu city (DGC) | 3.3 | 3.3 | 3.3 | 0.3 | 1.4 | 0.3 | 0.4 | 3.6 | 0.3 | 0.4 | 5.7 | 0.4 | 0.4 | 7.7 | 0.4 | -88.1% | 135.5% | -88.4% | 11.4% | | 113.9% | 11.8% |  |
| Daejeon city (DJC) | 0.6 | 0.6 | 0.6 | 0.1 | 0.5 | 0.1 | 0.2 | 1.2 | 0.2 | 0.2 | 2.0 | 0.2 | 0.3 | 2.7 | 0.2 | -56.1% | 368.4% | -59.6% | 38.9% | | 122.5% | 43.8% |  |
| Gangwon-do (GWP) | 5.3 | 5.3 | 5.3 | 6.0 | 9.9 | 16.0 | 5.8 | 18.0 | 47.0 | 5.6 | 27.4 | 83.2 | 5.5 | 37.6 | 122.7 | 3.8% | 604.5% | 2202.1% | -4.3% | | 108.5% | 161.3% |  |
| Gwangju city (GJC) | 8.1 | 8.1 | 8.1 | 8.1 | 17.4 | 7.8 | 6.4 | 30.8 | 6.5 | 5.2 | 45.1 | 5.6 | 5.3 | 55.7 | 6.0 | -34.3% | 590.0% | -25.4% | -17.6% | | 80.9% | -8.0% |  |
| Gyeonggi-do (GGP) | 0.1 | 0.1 | 0.1 | 0.1 | 0.4 | 0.1 | 0.1 | 1.1 | 0.1 | 0.1 | 1.7 | 0.1 | 0.0 | 2.4 | 0.0 | -55.6% | 2,544.4% | -55.6% | -20.0% | | 122.4% | -20.0% |  |
| Gyeongsangbuk-do (GBP) | 105.5 | 105.5 | 105.5 | 96.1 | 96.9 | 96.2 | 97.7 | 100.0 | 98.2 | 95.0 | 98.8 | 95.9 | 104.1 | 109.6 | 105.6 | -1.3% | 3.8% | 0.0% | 6.6% | | 9.5% | 7.5% |  |
| Gyeongsangnam-do (GNP) | 12.2 | 12.2 | 12.2 | 13.6 | 14.0 | 14.1 | 31.2 | 32.4 | 32.6 | 34.3 | 36.5 | 36.6 | 37.5 | 40.6 | 40.6 | 208.6% | 234.0% | 234.1% | 20.3% | | 25.1% | 24.6% |  |
| Incheon city (ICC) | 38.2 | 38.2 | 38.2 | 51.7 | 54.1 | 51.9 | 40.8 | 47.2 | 41.8 | 39.7 | 50.2 | 41.4 | 43.5 | 56.8 | 45.9 | 13.8% | 48.8% | 20.3% | 6.4% | | 20.4% | 10.0% |  |
| Jeju-do (JJP) | 5.1 | 5.1 | 5.1 | 4.3 | 4.8 | 4.4 | 4.1 | 5.4 | 4.5 | 4.0 | 6.2 | 4.7 | 4.0 | 7.1 | 5.1 | -21.5% | 38.1% | -1.4% | -2.0% | | 31.4% | 12.5% |  |
| Jeollabuk-do (JBP) | 2.0 | 2.0 | 2.0 | 1.9 | 2.0 | 1.7 | 1.2 | 1.6 | 1.1 | 0.6 | 1.3 | 0.6 | 0.7 | 1.7 | 0.6 | -67.0% | -16.0% | -69.5% | -44.1% | | 5.7% | -44.5% |  |
| Jeollanam-do (JNP) | 24.5 | 24.5 | 24.5 | 23.6 | 34.0 | 37.4 | 25.4 | 58.1 | 84.0 | 25.1 | 83.4 | 135.7 | 26.2 | 112.3 | 193.3 | 7.1% | 358.1% | 688.7% | 3.4% | | 93.4% | 130.2% |  |
| Sejong city (SJC) | 0.0 | 0.0 | 0.0 | 0.0 | 0.1 | 0.0 | 0.0 | 0.3 | 0.0 | 0.0 | 0.5 | 0.0 | 0.0 | 0.7 | 0.0 | - | - | - | - | | 161.5% | - |  |
| Seoul city (SUC) | 0.0 | 0.0 | 0.0 | 0.0 | 0.0 | 0.0 | 0.0 | 5.1 | 0.0 | 0.0 | 15.2 | 0.0 | 0.0 | 25.0 | 0.0 | - | - | - | - | | 389.4% | - |  |
| Ulsan city (USC) | 20.5 | 20.5 | 20.5 | 15.5 | 16.0 | 16.0 | 14.1 | 15.4 | 15.8 | 12.3 | 14.4 | 15.1 | 12.1 | 14.9 | 15.6 | -41.1% | -27.7% | -24.2% | -13.9% | | -3.3% | -1.5% |  |
| **Total** | **440.1** | **440.1** | **440.1** | **431.3** | **464.3** | **459.1** | **482.8** | **584.9** | **600.4** | **472.4** | **654.1** | **693.8** | **514.1** | **772.1** | **847.5** | **16.8%** | **75.4%** | **92.5%** | **6.5%** | | **32.0%** | **41.1%** |  |

**Supplementary Table S5.** Estimated costs for reducing of PM_10_ emissions by each scenario from 2010 to 2030. (unit: MEuro/year)

| Region | yr2010 | | | yr2015 | | | yr2020 | | | yr2025 | | | yr2030 | | | **((yr2030-yr2010)/yr2010)**  ***100** | | | | **((yr2030-yr2020)/yr2020)**  ***100** | | | |
| --- | --- | --- | --- | --- | --- | --- | --- | --- | --- | --- | --- | --- | --- | --- | --- | --- | --- | --- | --- | --- | --- | --- | --- |
| scenarios | BAU | AQ | MFR | BAU | AQ | MFR | BAU | AQ | MFR | BAU | AQ | MFR | BAU | AQ | MFR | **BAU** | **AQ** | **MFR** | **BAU** | | **AQ** | **MFR** |  |
| Busan city (BSC) | 1.2 | 1.2 | 1.2 | 1.1 | 0.0 | 1.5 | 1.0 | 0.2 | 2.2 | 1.0 | 0.3 | 2.8 | 0.9 | 2.0 | 3.3 | -24.2% | 57.3% | 164.5% | -8.7% | | 1200.0% | 48.4% |  |
| Chungcheongbuk-do (CBP) | 11.8 | 11.8 | 11.8 | 9.7 | 9.7 | 10.4 | 10.4 | 10.4 | 12.1 | 11.3 | 11.3 | 13.9 | 11.4 | 11.4 | 14.9 | -2.8% | -2.8% | 26.4% | 9.6% | | 9.6% | 22.5% |  |
| Chungcheongnam-do (CNP) | 608.0 | 608.0 | 608.0 | 595.3 | 596.8 | 598.9 | 723.9 | 727.4 | 734.9 | 704.2 | 710.3 | 723.2 | 771.9 | 781.0 | 799.4 | 26.9% | 28.5% | 31.5% | 6.6% | | 7.4% | 8.8% |  |
| Daegu city (DGC) | 7.0 | 7.0 | 7.0 | 1.0 | 1.3 | 1.3 | 1.3 | 1.8 | 1.9 | 1.9 | 2.4 | 2.7 | 2.0 | 2.6 | 2.9 | -72.3% | -63.5% | -58.7% | 48.9% | | 46.9% | 51.6% |  |
| Daejeon city (DJC) | 0.1 | 0.1 | 0.1 | 0.1 | 0.3 | 0.3 | 0.1 | 0.7 | 0.7 | 0.1 | 1.4 | 1.4 | 0.1 | 1.9 | 2.0 | -53.8% | 1,376.9% | 1,400.0% | 20.0% | | 182.4% | 182.6% |  |
| Gangwon-do (GWP) | 13.9 | 13.9 | 13.9 | 14.6 | 17.8 | 18.4 | 14.1 | 20.6 | 26.2 | 13.6 | 23.2 | 34.5 | 13.2 | 26.0 | 43.4 | -5.7% | 86.5% | 211.1% | -7.0% | | 26.2% | 65.5% |  |
| Gwangju city (GJC) | 38.8 | 38.8 | 38.8 | 39.6 | 42.2 | 43.3 | 29.5 | 34.6 | 37.2 | 21.8 | 28.2 | 32.1 | 22.9 | 30.9 | 36.7 | -41.0% | -20.5% | -5.6% | -22.4% | | -10.7% | -1.3% |  |
| Gyeonggi-do (GGP) | 0.3 | 0.3 | 0.3 | 0.2 | 0.2 | 0.2 | 0.2 | 0.2 | 0.3 | 0.1 | 0.3 | 0.3 | 0.1 | 0.3 | 0.4 | -57.1% | -10.7% | 25.0% | -25.0% | | 4.2% | 25.0% |  |
| Gyeongsangbuk-do (GBP) | 364.0 | 364.0 | 364.0 | 329.2 | 329.6 | 329.8 | 334.5 | 335.2 | 335.7 | 324.8 | 325.8 | 326.5 | 356.0 | 357.2 | 358.2 | -2.2% | -1.9% | -1.6% | 6.4% | | 6.6% | 6.7% |  |
| Gyeongsangnam-do (GNP) | 43.7 | 43.7 | 43.7 | 54.1 | 54.1 | 54.2 | 126.4 | 126.4 | 127.0 | 138.6 | 138.5 | 139.6 | 150.8 | 150.9 | 152.4 | 244.9% | 245.0% | 248.5% | 19.3% | | 19.4% | 20.0% |  |
| Incheon city (ICC) | 155.3 | 155.3 | 155.3 | 211.8 | 214.0 | 214.9 | 166.7 | 172.0 | 173.7 | 161.8 | 170.1 | 172.1 | 177.1 | 186.9 | 190.1 | 14.0% | 20.3% | 22.4% | 6.2% | | 8.6% | 9.5% |  |
| Jeju-do (JJP) | 15.1 | 15.1 | 15.1 | 11.9 | 12.9 | 13.2 | 11.3 | 13.1 | 14.5 | 11.1 | 13.8 | 16.2 | 11.1 | 14.6 | 18.0 | -26.3% | -3.6% | 19.1% | -1.0% | | 10.8% | 23.8% |  |
| Jeollabuk-do (JBP) | 13.2 | 13.2 | 13.2 | 12.3 | 13.0 | 13.0 | 7.7 | 8.5 | 8.5 | 3.9 | 4.5 | 4.5 | 4.3 | 5.1 | 5.1 | -67.8% | -61.2% | -61.1% | -45.0% | | -39.8% | -39.7% |  |
| Jeollanam-do (JNP) | 66.1 | 66.1 | 66.1 | 60.7 | 69.1 | 74.1 | 62.9 | 83.2 | 101.7 | 61.2 | 94.2 | 126.2 | 64.3 | 111.2 | 156.2 | -2.7% | 68.4% | 136.5% | 2.1% | | 33.7% | 53.6% |  |
| Sejong city (SJC) | 0.0 | 0.0 | 0.0 | 0.0 | 0.0 | 0.0 | 0.0 | 0.0 | 0.0 | 0.0 | 0.0 | 0.1 | 0.0 | 0.0 | 0.1 | - | - | - | - | | 0.0% | 133.3% |  |
| Seoul city (SUC) | 0.0 | 0.0 | 0.0 | 0.0 | 0.0 | 0.0 | 0.0 | 0.0 | 0.0 | 0.0 | 0.0 | 0.0 | 0.0 | 0.0 | 0.0 | - | - | - | - | | - | - |  |
| Ulsan city (USC) | 71.7 | 71.7 | 71.7 | 51.7 | 61.0 | 75.3 | 43.7 | 61.7 | 98.4 | 32.4 | 57.3 | 113.9 | 31.9 | 64.4 | 137.0 | -55.5% | -10.2% | 91.1% | -26.9% | | 4.4% | 39.2% |  |
| **Total** | **1,410.4** | **1,410.4** | **1,410.4** | **1,393.2** | **1,421.8** | **1,449.0** | **1,533.7** | **1,596.0** | **1,675.0** | **1,487.6** | **1,581.3** | **1,710.0** | **1,618.0** | **1,746.2** | **1,919.9** | **14.7%** | **23.8%** | **36.1%** | **5.5%** | | **9.4%** | **14.6%** |  |

**Supplementary Table S6.** Estimated costs for reducing of SO_2_ emissions by each scenario from 2010 to 2030. (unit: MEuro/year)
